# Effect of vitamin D, calcium, or combined supplementation on fall prevention: a systematic review and updated network meta-analysis

**DOI:** 10.1186/s12877-024-05009-x

**Published:** 2024-05-02

**Authors:** Long Tan, Ruiqian He, Xiaoxue Zheng

**Affiliations:** 1 Health Service Department, Guard Bureau of the General Office of the Central Committee of the Communist Party of China, Beijing, 100017 China; 2 Department of Health Care, Beijing Hospital, National Center of Gerontology, Institute of Geriatric Medicine, Chinese Academy of Medical Sciences, Dahua Road 1, Dongcheng District, Beijing, 100730 China

**Keywords:** Fall, Vitamin D, Network meta-analysis, Supplement

## Abstract

**Background:**

The association between vitamin D supplementation and the risk of falls in older adults has been controversial. This systematic review and network meta-analysis aims to assess the efficacy of vitamin D, calcium, and combined supplementation in the prevention of falls.

**Methods:**

Randomized controlled trials (RCTs) on the efficacy of vitamin D in fall prevention were systematically searched in PubMed, Embase, Cochrane Library, and Web of Science from inception to May 9, 2023. The network meta-analysis was performed using a random effects model in R4.1.3 and Stata15.0. Heterogeneity was evaluated by the *I*^*2*^ statistic, and publication bias was assessed using funnel plots, Begg’s test, and Egger’s tests. Data were pooled and expressed as relative risk (RR) and 95% confidence interval (CI).

**Results:**

A total of 35 RCTs involving 58,937 participants were included in this study, among which 11 RCTs (31.4%) applied calcium combined with vitamin D. There was low heterogeneity (*I*^*2*^ = 11%) among the included studies. Vitamin D supplementation at 800–1000 International Unit (IU)/d resulted in a lower risk of falls than placebo or no treatment (RR = 0.85, 95%CI: 0.74–0.95). In addition, 800–1000 IU/d of vitamin D with or without calcium were more effective in preventing falls than calcium alone. High-dose vitamin D (> 1000 IU/day) increased the risk of falls compared with 800–1000 IU/d of vitamin D. According to the subgroup analysis, daily administration of 800–1000 IU/d vitamin D was associated with a 22% reduction in the risk of falls (RR = 0.78, 95%CI:0.64–0.92), whereas intermittent vitamin D administration had no preventive effect. Furthermore, 800–1000 IU/d of vitamin D also significantly decreased the risk of falls in old adults with ≤ 50 nmol/L 25-hydroxyvitamin D [25(OH)D] (RR = 0.69, 95%CI:0.52–0.86) but not in individuals with > 50 nmol/L 25(OH)D.

**Conclusion:**

Vitamin D supplementation at 800–1000 IU/d is associated with a lower risk of falls among older adults. 800-1000IU/d of vitamin D has a benefit on prevention of falls in population received daily dose regimens and in population with vitamin D deficiency.

**Supplementary Information:**

The online version contains supplementary material available at 10.1186/s12877-024-05009-x.

## Background

Falls and fall-related injuries are common and potentially preventable causes of functional disability, morbidity, and increased health-care utilization among older individuals [1]. It was reported that one of every three individuals over 65 years of age has experienced at least one fall, with 5–6% of falls resulting in a fracture [2, 3]. Therefore, fall prevention is paramount for preventing fractures and reducing morbidity and mortality. Fall prevention guidelines have recommended vitamin D as a component of multifactorial interventions along with other strategies such as gait and balance training, home assessment and modifications, reduction or withdrawal of psychotropic drugs, treatment of impaired vision, management of postural hypotension, treatment of heart rate and abnormal heart rhythm, suitable footwear, and education [4, 5].

Vitamin D supplements are commonly taken to maintain bone health. The Bone Health and Osteoporosis Foundation (BHOF) recommends a daily intake of 800 to 1000 units of vitamin D for adults aged 50 years and older [6]. According to the Institute of Medicine (IOM) [7], the recommended daily intake of vitamin D is 600 International Unit (IU) for adults < 70 years of age and 800 IU for those ≥ 70 years of age.

However, previous randomized controlled trials (RCTs) have shown inconsistent effectiveness of vitamin D, calcium, and combined supplementation in fall prevention, which may be attributed to differences in vitamin D doses, mode of administration, and other regimen design features [8]. Previous systematic review and meta-analyses also had different recommendations. A recent meta-analysis reported that vitamin D supplementation had no impact on the incidence of fractures or falls nor clinically meaningful effects on bone mineral density [9]. On the other hand, Wu et al. found that vitamin D combined with calcium, but not vitamin D2 or D3 alone, significant lowered the risk of falls [10]. Similarly, Thanapluetiwong et al. [11] showed that vitamin D3 decreased the incidence of falls only when supplemented with calcium, but neither of the two articles conducted subgroup analysis of different vitamin D doses. Kong et al. revealed that 800–1000 IU/d of vitamin D was associated with lower risks of falls [12]. Ling et al. reported that combined supplementation of vitamin D (daily doses of 700–1000 IU) and calcium resulted in 12% reduction in the risk of falls [13]. Furthermore, Wei et al. found that 700–2000 IU/d of vitamin D was correlated with a lower risk of falls among ambulatory and institutionalized older adults [14].

With regard to higher doses of vitamin D, > 1000 IU/d of vitamin D supplementation resulted in an increased risk of first-time falls with fractures among community-dwelling older adults [15]. A daily dose of 2000 IU vitamin D in the VITAL trial failed to decrease the risk of falls in generally healthy adults [16].

Differences in vitamin D dosage, frequency of administration, and patient populations in the literature made it challenging to identify the best dose of vitamin D supplementation. A network meta-analysis (NMA) can pool the evidence from multiple RCTs through direct and indirect comparisons and thus provide a more comprehensive insight [17]. In the present study, we stratified subjects into various vitamin D dose groups and compared the risk of falls across different doses of vitamin D, calcium, and combined supplementation using NMA. We assigned probability ranking to each dosing regimen in order to identify the best concentration of vitamin D intake for older individuals aged 50 years and older.

## Methods

This study was performed in accordance with the Preferred Reporting Items for Systemic Reviews and Meta-Analyses (PRISMA) guidelines [18] (Table S1) and was registered on the international prospective system evaluation registration platform PROSPERO (CRD42023435299).

### Search strategy

Relevant studies published in English were systematically searched in PubMed, Embase, Cochrane Library, and Web of Science from inception to May 9, 2023 using the MeSH and free terms “vitamin D”, “ergocalciferol”, “accidental fall”, “fall” and “randomized controlled trial”. The literature search strategy is summarized in Table S2. The references of published systematic reviews were also manually searched to identify potential eligible studies.

### Eligibility criteria

Inclusion criteria: (1) RCTs published in English; (2) Vitamin D2 or D3 with or without calcium in the intervention groups (including daily, weekly, monthly or yearly intake); (3) Reported outcome data of falls; (4) Follow-up durations of at least three months. Exclusion criteria: (1) Animal or cell experiments, case reports, scientific experiment plans, reviews, letters, editorials, and conference abstracts; (2) Inaccessible full text; (3) Unextractable outcome data; (4) Combined with other therapies such as nutritional support, hormones, other medications, exercise training, or use of vitamin D analogues (e.g., calcitriol) or hydroxylated vitamin D; (5) If the same population was used in multiple studies, the studies with smaller dataset were excluded.

The abstract and full text of each study were independently screened by two reviewers (XXZ, LT) to determine eligibility, and any disagreements were resolved through discussion or by a third author (RQH).

### Data extraction

Data were extracted independently by two reviewers (XXZ and LT), including first author, year of publication, country, intervention and control measures, follow-up period, baseline serum 25-hydroxyvitamin D [25(OH)D] concentration, dwelling and study outcomes.

### Quality assessment

The quality of the included studies was independently evaluated by two researchers using the Cochrane risk of bias tool for randomized trials (RoB2) [19], and any disagreement was resolved by discussion with a third researcher. The RoB2 assessment has five domains (Table S3), namely bias arising from the randomization process, bias due to deviation from intended intervention, bias due to missing outcome data, bias in outcome measurement, and bias in selection of the reported result. Each domain is judged as “low risk of bias”, “high risk of bias”, and “some concerns”.

### Statistical analysis

The included studies varied in vitamin D dosage, frequency of administration and administration of calcium. To improve the mergeability of results, intermittent vitamin D intake was converted into daily intake by calculating the average dose per day. Based on the converted dose, subjects were divided into the ≤ 500 IU/d, 600–700 IU/d, 800-1000IU/d, 1100-1900IU/day, and ≥ 2000 IU/d groups. In addition, for vitamin D with calcium supplementation, subjects were classified based on whether or not calcium was also administered.

Data were analyzed by GeMTC and JAGS in R4.1.3. A Bayesian NMA was performed using the Markov Chain Monte Carlo (MCMC) methods [20, 21]. NMA is an extension of the standard meta-analysis that compares multiple treatments. Treatment effect can be evaluated by NMA using both direct and indirect comparisons. Due to variations among regimens, such as different dosages and various frequencies of administration, a standard random effects model was applied to provide more conservative estimations of effect size. Model convergence was performed using four Markov chains for simulation analysis with an initial value of 2.5 and 15,000 pre-simulated iterations for annealing, followed by 20,000 iterations. Model fit and global consistency were evaluated by the Deviation Information Criterion (DIC). The overall consistency between direct evidence and indirect evidence was analyzed using the consistent and inconsistent DIC values, respectively [22]. A difference in DIC of < 5 indicates no inconsistency, and the consistency model is fitted; otherwise, the inconsistency model is fitted. If there is a closed-loop network, local consistency was analyzed using a node splitting method [23]. A *P* < 0.05 indicates local inconsistency. Heterogeneity among studies was evaluated by the I^2^ statistic, and a value of > 50% indicates significant heterogeneity. Publication bias was assessed by comparison-adjusted funnel plots, Begg’s rank correlation test, and Egger’s test. Sensitivity analyses were conducted on studies without high risk of bias. Post-hoc subgroup analyses were also performed by various factors including gender, dwelling, dosing frequency of vitamin D (daily and intermittent), and baseline 25(OH)D concentrations. Categorical variables are expressed as risk ratios (RR) and 95% confidence interval (CI). If the value of “1” is not included in the 95% CI, the difference is considered statistically significant.

The efficacy of all treatment regimens was simultaneously assessed using a Bayesian framework-based random effects model. Network relationship graphs for outcome indicators, cumulative probability ranking graphs, league tables, and “comparison-corrected” funnel plots were generated. The effects of each intervention were estimated, ranked, and clustered based on the surface under the cumulative ranking curve (SUCRA), and the quality of intervention measures was ranked according to the SUCRA value. The SUCRA represents the percent of efficacy or safety achieved by an agent compared to an imaginary agent that is always the best without uncertainty (e.g., SUCRA = 100%). The SUCRA score is a percentage that ranges from 0 to 100%, and a score closer to 100% indicates a more effective intervention [24]. The NMA was completed using R4.1.3 and Stata 15.0.

## Results

### Study search and characteristics

**Fig. 1:**
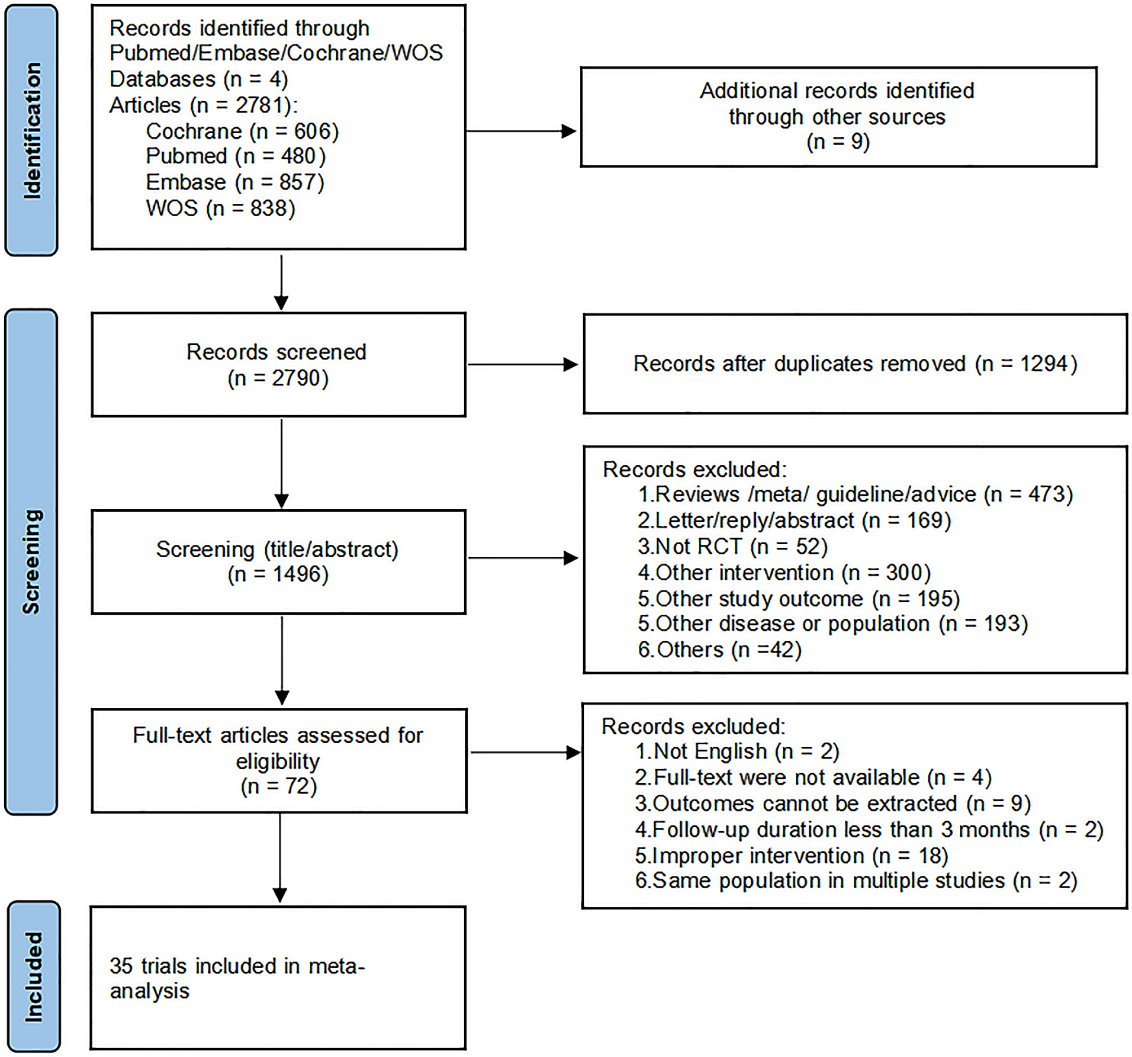
PRISMA flow diagram of study selection

We initially identified 2790 studies, of which 1496 were removed due to duplication, 1424 were excluded after initial review of the title and abstract, and 72 studies were retrieved for full-text review. A final total of 35 eligible studies were included in this meta-analysis. The specific screening process is shown in Fig. 1.

The 35 studies [25–59] involving 58,937 patients were conducted in Europe (*n* = 19), Australia (*n* = 7), North America (*n* = 6), Asia (*n* = 2) and South America (*n* = 1). The mean age of the patients varied from 59 to 89 years of age. Calcium and vitamin D combined supplementation was used in 11 studies(31.4%), 800–1000 IU/d of vitamin D in 19 studies, < 800 IU/d of vitamin D in 6 studies, and > 1000 IU of vitamin D in 18 studies. The characteristics of the included studies are summarized in Table 1.

**Table 1:** The essential characteristics of the included studies

Source	Country	Numbers	Study Length	Wome *n* (%)	Age	Treatment	25-OH-VD (nmol/L)	Dwelling	Fallers/total
Graafmans,1996 [25]	The Netherlands	354	7 months	85%	83	E: 400 IU VD_3_/d C: Placebo	ND	In homes for elderly	E: 62/177 C: 65/177
Pfeifer,2000 [26]	Germany	148	1 year	100%	74	E:800IU Cholecalciferol + 1200 mg elemental calcium C: placebo + 1200 mg elemental calcium	E: 25.7(13.6) C: 24.6(12.1)	Ambulatory	E: 11/74 C: 19/74
Chapuy,2002 [27]	France	583	2 years	100%	85.2	E: 800IU Cholecalciferol + 1200 mg elemental calcium C: Placebo	E: 21.8(14.9) C: 22.7(17.2)	In apartment for elderly	E: 251/393 C: 118/190
Bischoff,2003 [28]	Switzerland	122	12 weeks	100%	85.3	E:800IU Cholecalciferol + 1200 mg calcium carbonate C: 1200 mg calcium carbonate	E: 30.7 C: 28.95	In long stay geriatric care	E: 14/62 C: 18/60
Trivedi,2003 [29]	UK	2686	5 years	24.2%	74.8	E: 100 000IU Cholecalciferol every 4 months C: Placebo	ND	Community dwelling	E: 254/1345 C: 261/1341
Dhesi,2004 [30]	UK	139	6 months	77.7%	76.8	E: 600000IU of ergocalciferol im once C: Placebo	E: 26.7 C: 25	Community dwelling	E: 11/70 C: 14/69
Harwood,2004 [31]	UK	150	1 year	100%	81.2	E1: 800IU Cholicalciferol + 1 g calcium E2: 300000IU VD_2_ im once + 1 g elemental calcium E3: 300000IU VD_2_ im once C: No treatment(no placebo)	E1: 29 E2: 30 E3: 28 C: 30	In rehabilitation wards	E1: 7/39 E2: 6/36 E3: 2/38 C: 13/37
Flicker,2005 [32]	Australia	625	2 years	94.9%	83.4	E: ergocalciferol 10000IU once weekly and then 1000IU/d + 600 mg elemental calcium C: 600 mg elemental calcium daily	E: 25–90 C: 25–90	Nursing home + Hostel	E: 170/313 C: 185/312
Porthouse,2005 [33]	UK	3314	12 months	100%	76.8	E: VD_3_ 800 IU + 1000 mg calcium (calcium carbonate) C: control	ND	Community dwelling	E:329/1321^a^ C:561/1993^a^
Bischoff-Ferrari,2006 [34]	USA	445	3 years	55.3%	70.8	E: 700 IU Cholecalciferol + 500 mg calcium citrate malate C: Placebo	E: 75(35) C: 72(33)	Community dwelling	E: 107/219 C: 124/226
Law,2006 [35]	UK	3717	10 months	76%	85	E: 2.5 mg (= 1100 IU/d) ergocalciferol every 3 months C: No treatment(no placebo)	E: 47 C: ND	In residential care homes	E: 770/1762 C: 833/1955
Smith,2007 [36]	UK	9440	3 years	53.9%	79.1	E: 300000IU intramuscular VD2/year C: Placebo	141(59.2)	Community dwelling	E:2544/4727 C:2577/4713
Broe,2007 [37]	USA	124	5 months	72.6%	89	E1: 200 IU VD_2_/d E2: 400 IU VD_2_/d E3: 600 IU VD_2_/d E4: 800 IU VD_2_/d C: Placebo	E1: 44.4(23) E2: 51.7(29) E3: 41.2(19) E4: 53.4(23) C: 52.9(28)	Nursing home residents	E1: 15/26 E2: 15/25 E3: 15/25 E4: 5/23 C: 11/25
Prince,2008 [38]	Australia	302	1 year	100%	77.2	E: 1000IU Ergocalciferol + 1000 mg/d calcium citrate C: Placebo + 1000 mg calcium citrate	E: 45.2(12.5) C: 44.2(12.7)	Community dwelling	E: 80/151 C: 95/151
Pfeifer,2009 [39]	Germany Austria	242	20 months	74.8%	77	E: 800IU cholecalciferol + 1000 mg elemental calcium C: Placebo + 1000 mg elemental calcium	E: 55(18) C: 54(18)	Community dwelling	E: 49/121 C: 75/121
Kärkkäinen,2010 [40]	Finland	3139 (593 subsample participants)	3 years	100%	67.4	E: 800 IU cholecalciferol + 1,000 mg calcium carbonate C: control without placebo	E: 50.1(18.8) C: 49.2(17.7)	Community dwelling	E: 179/287 C: 205/306
Sanders,2010 [41]	Australia	2256	3–5 years	100%	76.1	E: A single oral dose of 500,000 IU cholecalciferol in autumn or winter C: Placebo	E: 53 C: 45	Community dwelling	E: 837/1311 C: 769/1125
Witham,2010 [42]	UK	105	20 weeks	34.3%	79.7	E: 100000IU D_2_ oral at baseline and 10w C: Placebo	E: 20.5(8.9) C: 23.7(10)	Primary and secondary care	E: 2/53 C: 5/52
Glendenning,2012 [43]	Australia	686	9 months	100%	76.7	E: 150,000 IU oral cholecalciferol every 3 months + 1300 mg calcium/d C: Placebo + 1300 mg calcium/d	E: 65.0 (17.8) C: 66.5(27.1)	Community dwelling	E: 102/353 C: 89/333
Witham,2013 [44]	UK	159	12 months	48.4%	76.8	E: 100,000 IU oral cholecalciferol every 3 months C: Placebo	E: 44.9(15) C: 44.9(15)	Community dwelling	E: 25/80 C: 26/79
Wood,2014 [45]	UK	305	1 year	100%	63.8	E1: 400IU VD_3_ E2: 1000IU VD_3_ C: Placebo	33.8	Community dwelling	E1: 33/102 E2: 27/101 C: 31/102
Houston,2015 [46]	USA	68	5 months	72.1%	77.9	E: two VD_3_ 50,000 IU capsules /month; C: Placebo	E: 56.2 (30.5) C: 47.2 (26.5)	Community dwelling	E: 11/38 C: 12/30
Hansen,2015 [47]	USA	230	12 months	100%	61	E1: 800 IU VD_3_ daily E2: loading dose (50,000 IU daily for 15 days), then twice monthly 50,000 IU VD_3_ C: Placebo	52.4(7.5)	Community dwelling	E1: 24/75 E2: 22/79 C: 23/76
Uusi-Rasi,2015 [48]	Finland	409	2 years	100%	74.2	E: 800IU VD_3_ without exercise C: Placebo without exercise	E: 65.9(17.2) C: 67.6(18.7)	Home dwelling	E: 66/102 C: 75/102
Cangussu,2016 [49]	Brazil	160	9 months	100%	59	E: 1000IU VD_3_ C: Placebo	E: 37.4(18.7) C: 42.2(16.7)	Ambulatory	E: 19/80 C: 37/80
Imaoka,2016 [50]	Japan	91	9 months	75.8%	84.8	E: 900IU VD_3_ C: no treatment	E: 35.2(13.2) C: 28.2(11)	Institutional care facility	E: 6/23 C: 9/23
Jin,2016 [51]	Australia	413	24 month	50.4%	63.2	E: oral 50000IU VD_3_ monthly C: Placebo	E: 43.7(11.8) C: 43.8(12.7)	Community dwelling	E: 2/209 C:0/204
Bischoff-Ferrari,2016 [52]	Switzerland	200	12 months	67%	78	E: 60000IU VD_3_/month C: 24000IU VD_3_/month	E: 52.2(23) C: 46.7(24.5)	Community dwelling	E: 45/67 C: 32/67
Levis,2017 [53]	USA	130	9 months	0%	72.4	E: 4,000 IU cholecalciferol daily C: Placebo	E: 57.7(12.5) C: 56.2(13.2)	Ambulatory	E: 8/66 C: 11/64
Hin,2017 [54]	UK	305	1 year	49.2%	71.6	E1: 2000IU VD_3_/d E2: 4000IU VD_3_/d C: Placebo	50(18)	Community dwelling	E: 34/204 C: 14/101
Khaw,2017 [55]	New Zealand	5108	3.4 years	41.8%	65.9	E: An initial oral dose of 200 000 IU colecalciferol followed by monthly 100 000 IU colecalciferol C: Placebo	63(24)	Ambulatory	E: 1312/2558 C: 1326/2550
Asprey,2019 [56]	England	379	12 months	48%	75	E1: 24000IU VD_3_/month E2: 48000IU VD _3_/month C: 12000IU VD_3_/month	E1: 39.5(20.6) E2: 38.9(19.7) C: 41.6(19.9)	Community dwelling	E1: 43/125 E2: 50/128 C: 48/126
Prithiani,2021 [57]	Pakistan	400	24 months	52.8%	61.5	E: 100,000 IU VD_3_ oral monthly C: Placebo	E: 56(14.5) C: 58 (14)	In hospital	E: 42/170 C: 43/173
Waterhouse,2021 [58]	Australia	21,315 (2200 diary participants)	5 years	46%	69.3	E: oral 60,000 D_3_ monthly C: Placebo	ND	ND	E: 159/1109 C: 153/1091
Appel,2021 [59]	USA	688	22 months	43.6%	77.2	E1: 1000IU VD_3_/d E2: 2000IU VD_3_/d E3: 4000IU VD_3_/d C: 200IU VD_3_/d	55.3	Community dwelling	E1: 43/121 E2: 41/68 E3: 41/69 C: 123/256

*Note* ND, no data; Data are expressed as mean(SD), mean or n (%). E, Experimental group; C, Control group; a, data obtained from previous meta-analysis

### Quality and risk of bias assessment

**Fig. 2:**
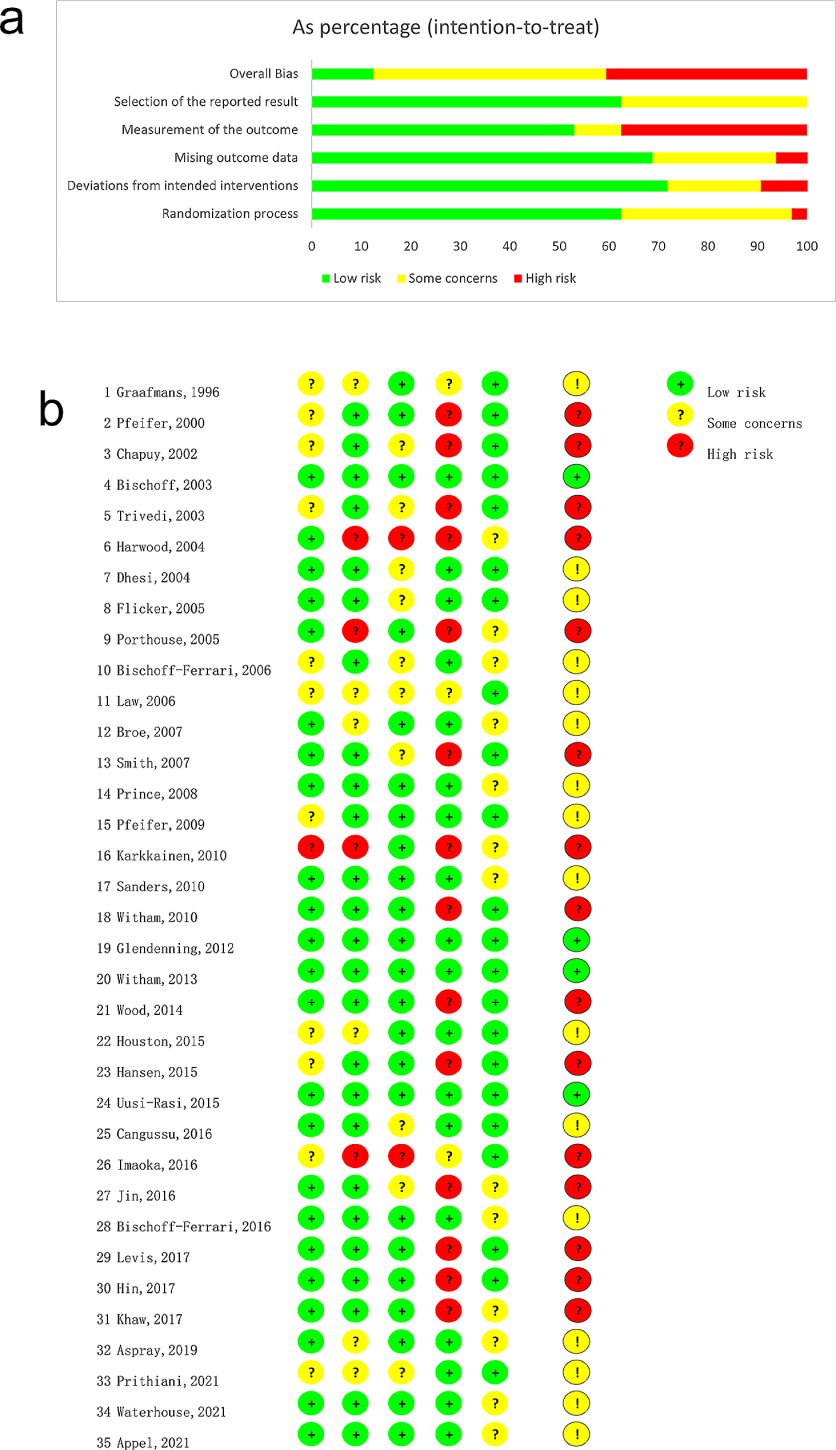
Summary of the risk of bias (using RoB2) in the included RCTs. (**a**) Results of each risk of bias item, presented as a percentage of included studies. (**b**) Results of the risk of bias in the 35 included trials

As shown in Fig. 2, the risk of bias arising from the randomization process was high in one studies, of some concerns in 11 studies due to the lack of random allocation sequence concealment, and low in the remaining studies. For biases due to deviations from intended interventions, 4 studies had high risk, 6 studies had some concerns, and the remaining studies had low risk. For biases due to missing outcome data, 2 studies had high risk and 10 studies had some concerns. For biases in measurement of the outcome, 14 studies had high risk and 3 studies had some concerns and the remaining had low risk. Lastly, for biases in selection of the reported results, 13 studies had some concerns and the remaining had low risk. Overall, twenty studies(57.1%) were rated as having low-to-moderate risk of bias.

### Bayesian NMA

#### Network plot

**Fig. 3:**
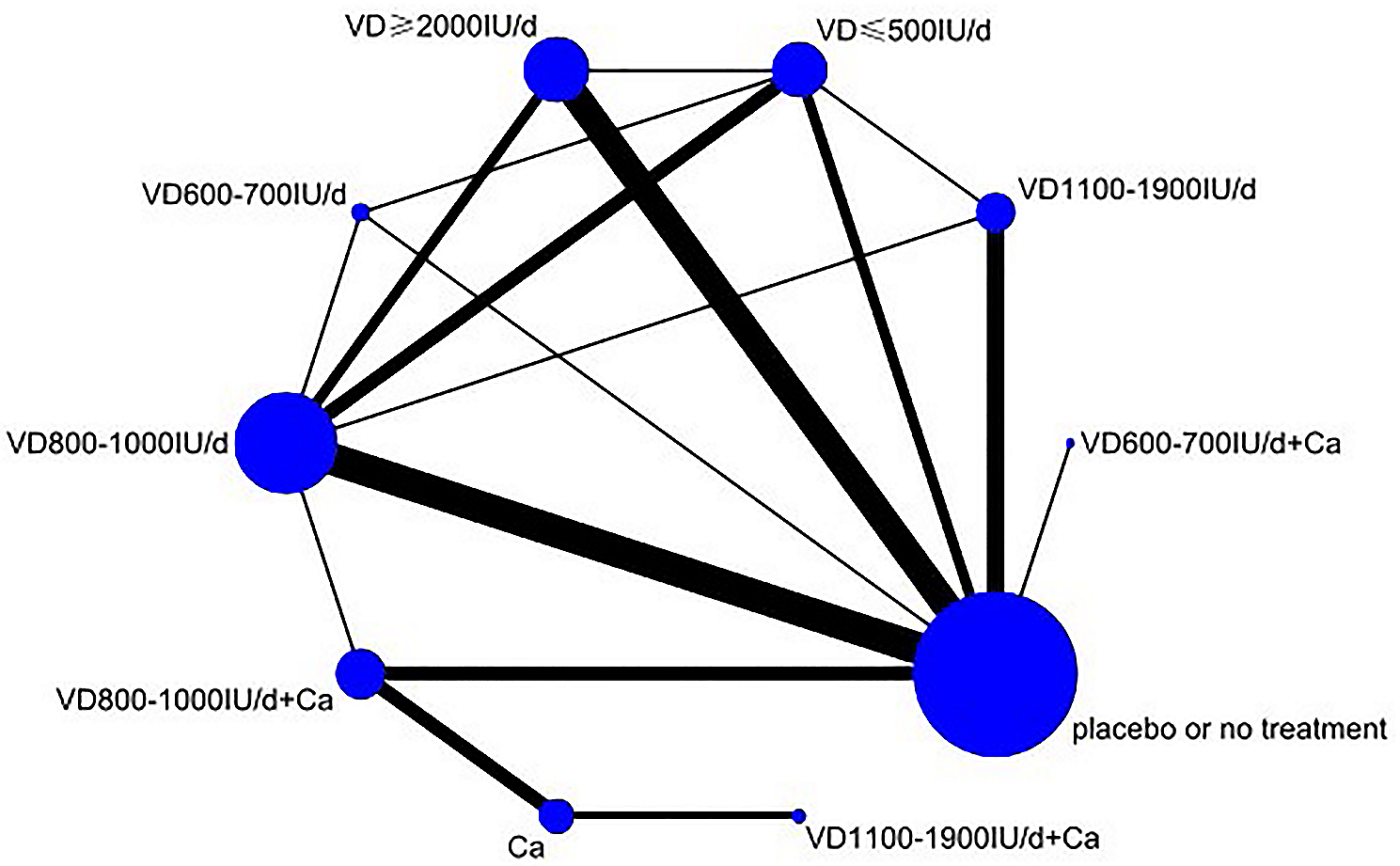
Network meta-analysis maps of fall prevention effects of different vitamin D supplementation regimens

The network plot for the effectiveness of vitamin D supplementation in fall prevention is shown in Fig. 3. Directly comparable treatments are connected by a line, and the thickness of the line is proportional to the number of studies compared in pairs. The diameter of the circle is proportional to the number of participants who received the intervention measures.

#### Outcomes

**Fig. 4:**
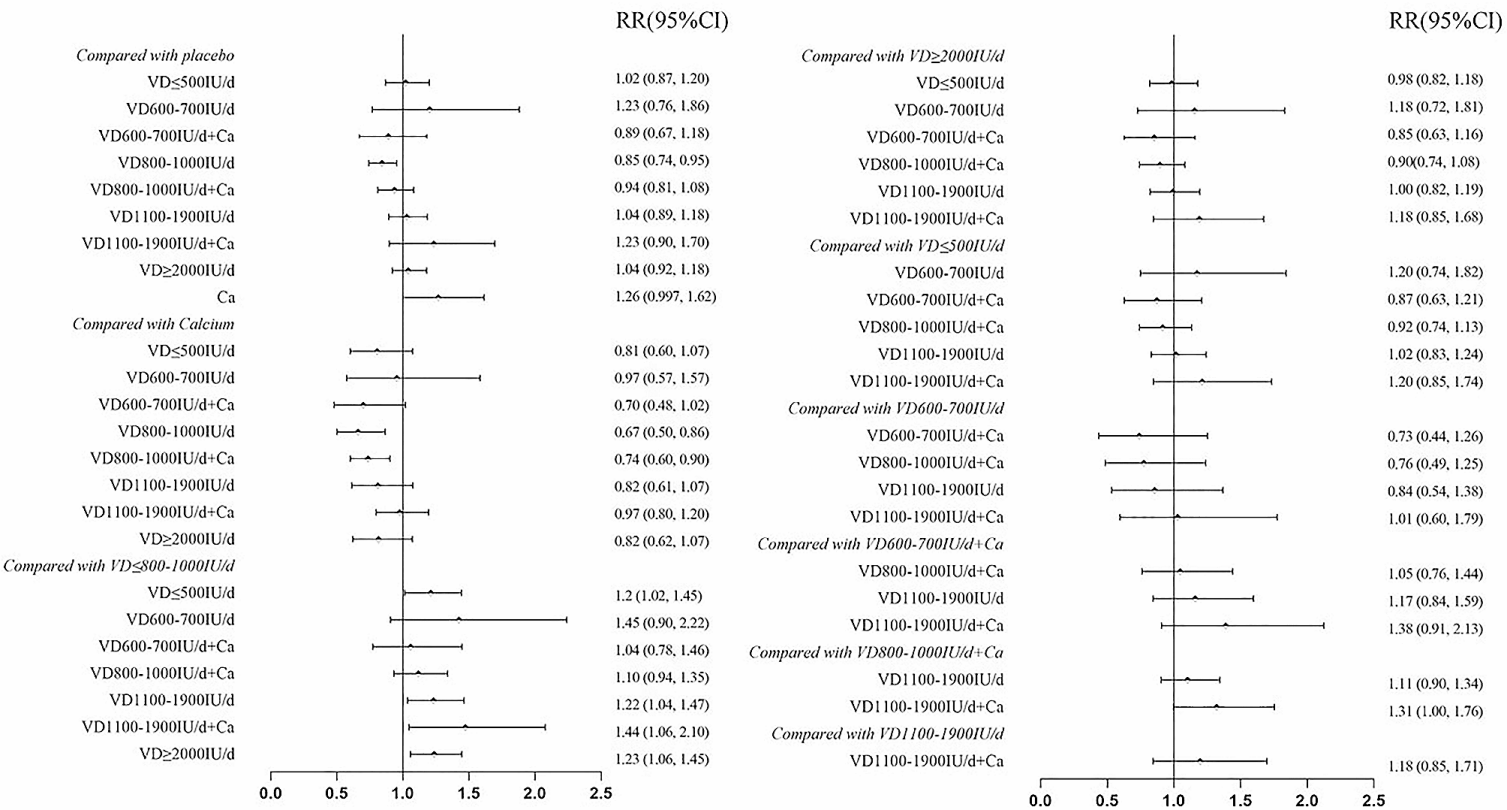
Forest plot of the risk of falls

There was no significant heterogeneity (I^2^ = 11%) among the included studies. The pooled results of 35 RCTs showed that 800–1000 IU/d of vitamin D significantly lowered the risk of falls compared to the placebo or no treatment (RR = 0.85, 95%CI: 0.74–0.95) (Table 2; Fig. 4). In addition, 800–1000 IU/d of vitamin D with or without calcium also decreased the frequency of falls compared with calcium alone (RR = 0.74, 95%CI: 0.60–0.90; RR = 0.67, 95%CI: 0.50–0.86; respectively). However, vitamin D supplementation at doses of ≤ 500 IU/d (RR = 1.2, 95%CI: 1.02–1.45), 1100–1900 IU/d (RR = 1.22, 95%CI: 1.04–1.47), 1100–1900 IU/d + Ca (RR = 1.44, 95%CI: 1.06–2.1), and ≥ 2000 IU/d (RR = 1.23, 95%CI: 1.06–1.45) resulted in significantly increased frequency of falls compared with 800–1000 IU/d of vitamin D. Other vitamin D doses showed no significant impact on the risk of falls.

**Table 2:** League table of treatment comparisons

Intervention	VD800-1000IU/d	VD800-1000IU/d + Ca	VD600-700IU/d + Ca	Placebo or no treatment	VD ≤ 500IU/d	VD600-700IU/d	VD1100-1900IU/d	VD1100-1900IU/d + Ca	VD ≥ 2000IU/d	Ca
VD800-1000IU/d		1.1 (0.94, 1.35)	1.04 (0.78, 1.46)	**1.18 (1.06, 1.35)**	**1.2 (1.02, 1.45)**	1.45 (0.9, 2.22)	**1.22 (1.04, 1.47)**	**1.44 (1.06, 2.1)**	**1.23 (1.06, 1.45)**	**1.49 (1.16, 2)**
VD800-1000IU/d + Ca	0.91 (0.74, 1.07)		0.95 (0.69, 1.31)	1.07 (0.93, 1.23)	1.09 (0.88, 1.35)	1.31 (0.8, 2.04)	1.11 (0.9, 1.34)	1.31 (1, 1.76)	1.11 (0.92, 1.34)	**1.35 (1.12, 1.66)**
VD600-700IU/d + Ca	0.96 (0.69, 1.28)	1.05 (0.76, 1.44)		1.12 (0.85, 1.49)	1.15 (0.83, 1.59)	1.38 (0.79, 2.29)	1.17 (0.84, 1.59)	1.38 (0.91, 2.13)	1.17 (0.86, 1.59)	1.42 (0.98, 2.08)
Placebo or no treatment	**0.85 (0.74, 0.95)**	0.94 (0.81, 1.08)	0.89 (0.67, 1.18)		1.02 (0.87, 1.2)	1.23 (0.76, 1.86)	1.04 (0.89, 1.18)	1.23 (0.9, 1.7)	1.04 (0.92, 1.18)	1.26 (0.997, 1.62)
VD ≤ 500IU/d	**0.83 (0.69, 0.98)**	0.92 (0.74, 1.13)	0.87 (0.63, 1.21)	0.98 (0.83, 1.15)		1.2 (0.74, 1.82)	1.02 (0.83, 1.24)	1.2 (0.85, 1.74)	1.02 (0.85, 1.22)	1.24 (0.93, 1.67)
VD600-700IU/d	0.69 (0.45, 1.12)	0.76 (0.49, 1.25)	0.73 (0.44, 1.26)	0.81 (0.54, 1.32)	0.83 (0.55, 1.34)		0.84 (0.54, 1.38)	1.01 (0.6, 1.79)	0.85 (0.55, 1.39)	1.03 (0.64, 1.77)
VD1100-1900IU/d	**0.82 (0.68, 0.96)**	0.9 (0.74, 1.11)	0.86 (0.63, 1.19)	0.96 (0.84, 1.12)	0.98 (0.81, 1.21)	1.18 (0.72, 1.84)		1.18 (0.85, 1.71)	1 (0.84, 1.21)	1.22 (0.93, 1.64)
VD1100-1900IU/d + Ca	**0.69 (0.48, 0.95)**	0.76 (0.57, 1)	0.72 (0.47, 1.1)	0.81 (0.59, 1.11)	0.83 (0.58, 1.17)	0.99 (0.56, 1.67)	0.84 (0.59, 1.17)		0.85 (0.59, 1.18)	1.03 (0.84, 1.25)
VD ≥ 2000IU/d	**0.81 (0.69, 0.94)**	0.9 (0.74, 1.08)	0.85 (0.63, 1.16)	0.96 (0.85, 1.09)	0.98 (0.82, 1.18)	1.18 (0.72, 1.81)	1 (0.82, 1.19)	1.18 (0.85, 1.68)		1.21 (0.93, 1.61)
Ca	**0.67 (0.5, 0.86)**	**0.74 (0.6, 0.9)**	0.7 (0.48, 1.02)	0.79 (0.62, 1.003)	0.81 (0.6, 1.07)	0.97 (0.57, 1.57)	0.82 (0.61, 1.07)	0.97 (0.8, 1.2)	0.82 (0.62, 1.07)	

Numbers in each cell represent the RR (95%CI) for falls between the treatment specified in the column versus that specified in the row

#### SUCRA ranking

**Fig. 5:**
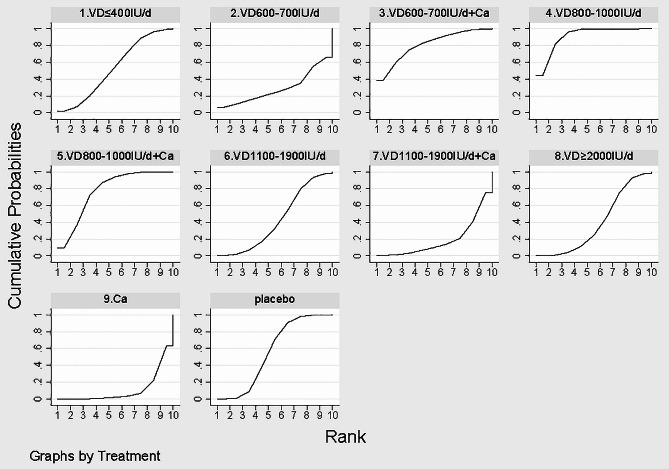
Comparison of the effectiveness of different interventions based on the surface under the cumulative ranking curves (SUCRA). *Note* In this graphical approach, rankings are made based on the area under the curve (AUC). The greater the AUC, the higher the likelihood that an intervention is in the top rank or one of the top ranks

Cumulative probability ranking revealed that 800–1000 IU/d of vitamin D (SUCRA: 91.1%), 600–700 IU/d vitamin D with calcium (SUCRA: 80.8%), and 800–1000 IU/d of vitamin D with calcium (SUCRA: 77.3%) may be the best three regimens for lowering the risk of falls (Fig. 5).

#### Publication bias and local inconsistency

**Fig. 6:**
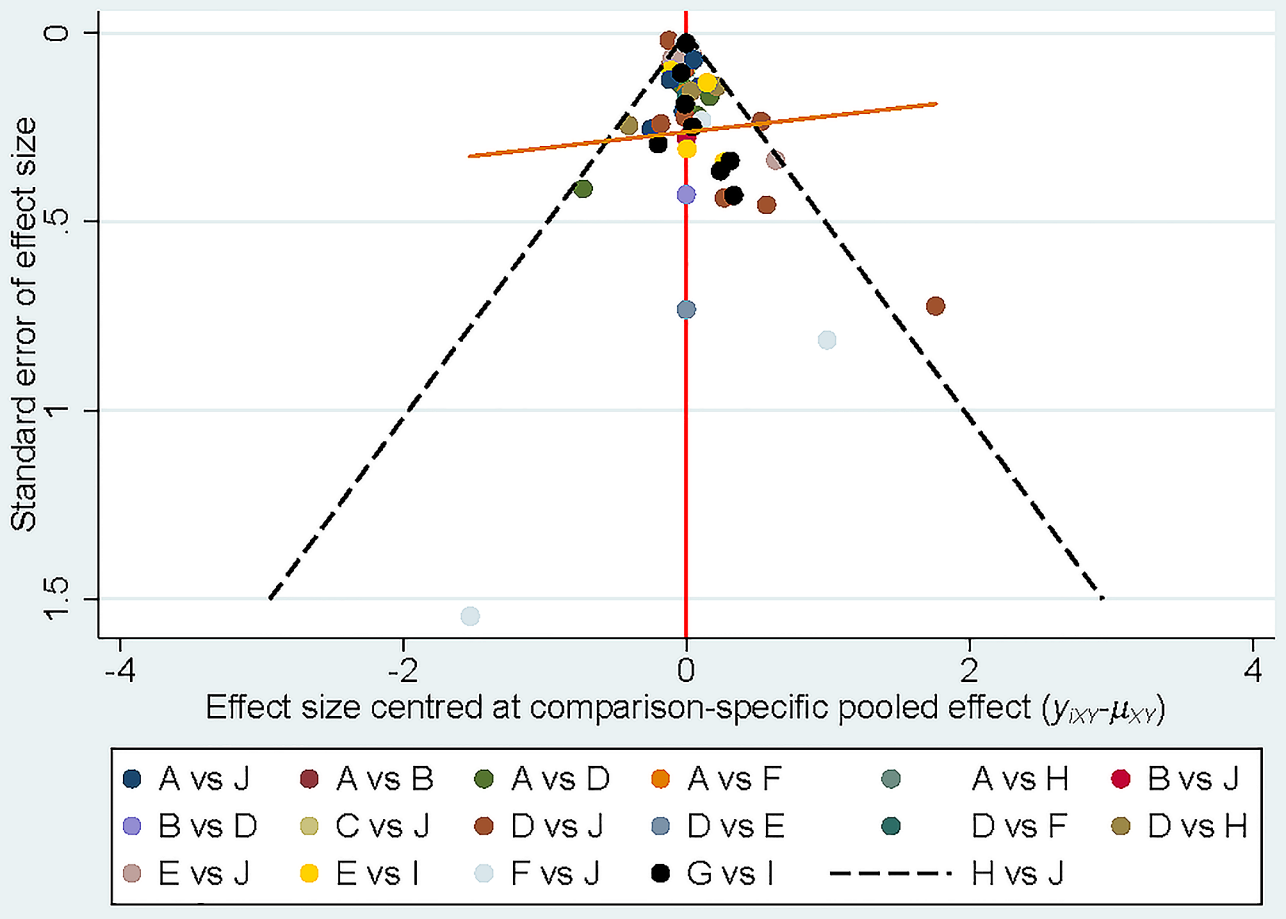
Comparison-corrected funnel plot. A, ≤ 500 IU/d vitamin D; B, 600–700 IU/d vitamin D; C, 600-700IU/d vitamin D + Ca; D, 800–1000 IU/d vitamin D; E, 800–1000 IU/d vitamin D + Ca; F, 1100–1900 IU/d vitamin D; G, 1100–1900 IU/d vitamin D + Ca; H, ≥ 2000 IU/d vitamin D; I, Calcium; J, Placebo or no treatment

The comparison-corrected funnel plot was roughly symmetrical (Fig. 6). However, the Begg’s and Egger’s tests indicated some publication bias in the studies (*P* = 0.7 Begg’s test, *P* = 0.033 Egger’s test).

We performed a node-splitting analysis to determine the consistency in any closed loops of two interventions. Inconsistency was present in comparisons of ≥ 2000 IU/d vitamin D with placebo or no treatment (*P* = 0.01). Compared with ≥ 2000 IU/d of vitamin D, the RR (95%CI) of the placebo or no treatment group was 1.02 (95%CI: 0.92–1.14) according to direct comparison, 0.73 (95%CI:0.59–0.91) according to indirect comparison, and 0.96 (95%CI:0.85–1.09) according to the overall result. Consistency in direct and indirect estimates was detected (*P* > 0.05) in all other closed loops.

#### Subgroup analysis

**Fig. 7:**
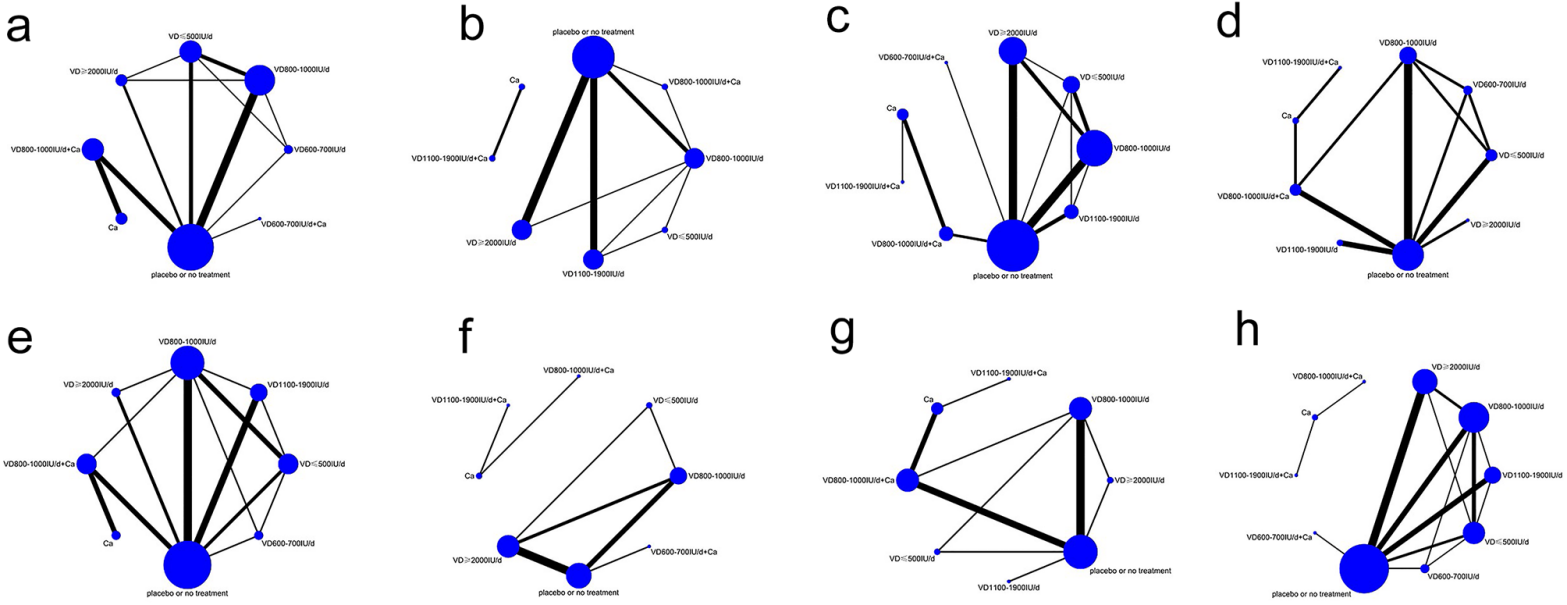
Network meta-analysis maps of subgroup analyses. (**a**) Daily dosing regimens; (**b**) Intermittent dosing regimens; (**c**) Ambulatory and community-dwelling elderly; (**d**) Institution-dwelling elderly; (**e**) Mean baseline 25(OH)D concentration ≤ 50 nmol/L; (**f**) Mean baseline 25(OH)D concentration > 50 nmol/L; (**g**) Female subjects only; (**h**) Both male and female subjects

As shown in Fig. 7, we performed subgroup NMA by various factors including gender, dwelling, dosing frequency of vitamin D (daily and intermittent), and baseline 25(OH)D concentrations.

##### Subgroup analysis of vitamin D dosing frequency

In 19 studies that applied daily dosing (Fig. 7a), 800–1000 IU/d of vitamin D significantly lowered the risk of falls (by 22%) compared with placebo or no treatment (RR = 0.78, 95%CI:0.64–0.92), calcium alone (RR = 0.63, 95%CI:0.45–0.83), ≤ 500 IU/d vitamin D (RR = 0.74, 95%CI:0.58–0.91), and ≥ 2000 IU/d vitamin D (RR = 0.63, 95%CI:0.48–0.83). In addition, 800–1000 IU/d vitamin D combined with calcium resulted in lower risk of falls compared with calcium alone (RR = 0.74, 95%CI:0.6–0.9)(Table S4, Fig. S1). Of the 18 studies that applied intermittent dosing (Fig. 7b), NMA was performed on 16 studies since the other two studies were unable to form a network structure with other studies. No significant differences in the risk of falls were detected among different supplementation regimens (Fig. S2).

##### Subgroup analysis of dwelling

In 24 studies involving ambulatory and community-dwelling elderly individuals (Fig. 7c), 800–1000 IU/d of vitamin D significantly lowered the risk of falls compared with placebo or no treatment (RR = 0.88, 95%CI:0.76–0.98), calcium alone (RR = 0.71, 95%CI:0.5–0.96), and ≥ 2000 IU/d of vitamin D (RR = 0.83, 95%CI:0.69–0.98). Furthermore, 800–1000 IU/d of vitamin D combined with calcium led to reduced risk of falls compared with calcium alone (RR = 0.74, 95%CI:0.58–0.91)(Table S5, Fig. S3). In studies with institution-dwelling elderly (Fig. 7d), the width of the CI was wide due to limited number of studies (Table S6, Fig. S4). Nonetheless, the risk of falls was significantly lower in the 800–1000 IU/d vitamin D group than in the placebo or no treatment (RR = 0.40, 95%CI:0.16–0.88) and ≤ 500 IU/d of vitamin D (RR = 0.37, 95%CI:0.12–0.99) groups. No significant differences in the risk of falls were detected among other regimens.

##### Subgroup analysis of baseline 25(OH)D concentration

In the 18 studies with a mean baseline 25(OH)D concentration of ≤ 50 nmol/L(Fig. 7e), 800–1000 IU/d of vitamin D significantly lowered the risk of falls compared with placebo or no treatment (RR = 0.69, 95%CI:0.52–0.86), calcium alone (RR = 0.57, 95%CI:0.34–0.85), ≤ 500 IU/d of vitamin D (RR = 0.67, 95%CI:0.49–0.88), 600–700 IU/d of vitamin D (RR = 0.57, 95%CI:0.34–0.98), 800–1000 IU/d of vitamin D plus calcium (RR = 0.72, 95%CI:0.5–0.98), 1100–1900 IU/d of vitamin D ( RR = 0.67, 95%CI:0.5–0.91), and ≥ 2000 IU/d of vitamin D (RR = 0.7, 95%CI:0.48–0.97)(Table S7, Fig. S5). Of the 11 studies with a mean baseline 25(OH)D concentration > 50 nmol/L(Fig. 7f), NMA was only performed on 9 studies since two studies were unable to form a network structure with other studies. The width of CIs was wide and no significant differences were detected among the different supplementation regimens (Fig. S6).

##### Subgroup analysis of gender

Among the 13 studies that only included females (Fig. 7g), 800–1000 IU/d of vitamin D decreased the risk of falls by 22% compared with placebo or no treatment (RR = 0.78, 95%CI:0.55–0.97). There were no significant differences in risk of falls between higher doses of vitamin D and 800–1000 IU/d of vitamin D (Table S8 and Fig. S7). Of the 21 studies that enrolled both male and female subjects (Fig. 7h), NMA was performed on 19 studies since two studies were unable to form a network structure with other studies. Our results showed that 800–1000 IU/d of vitamin D significantly lowered the risk of falls compared with placebo or no treatment (RR = 0.86, 95%CI:0.7–0.99) and ≥ 2000 IU/d of vitamin D ( RR = 0.8, 95%CI:0.64–0.95) (Table S9 and Fig. S8).

**Fig. 8:**
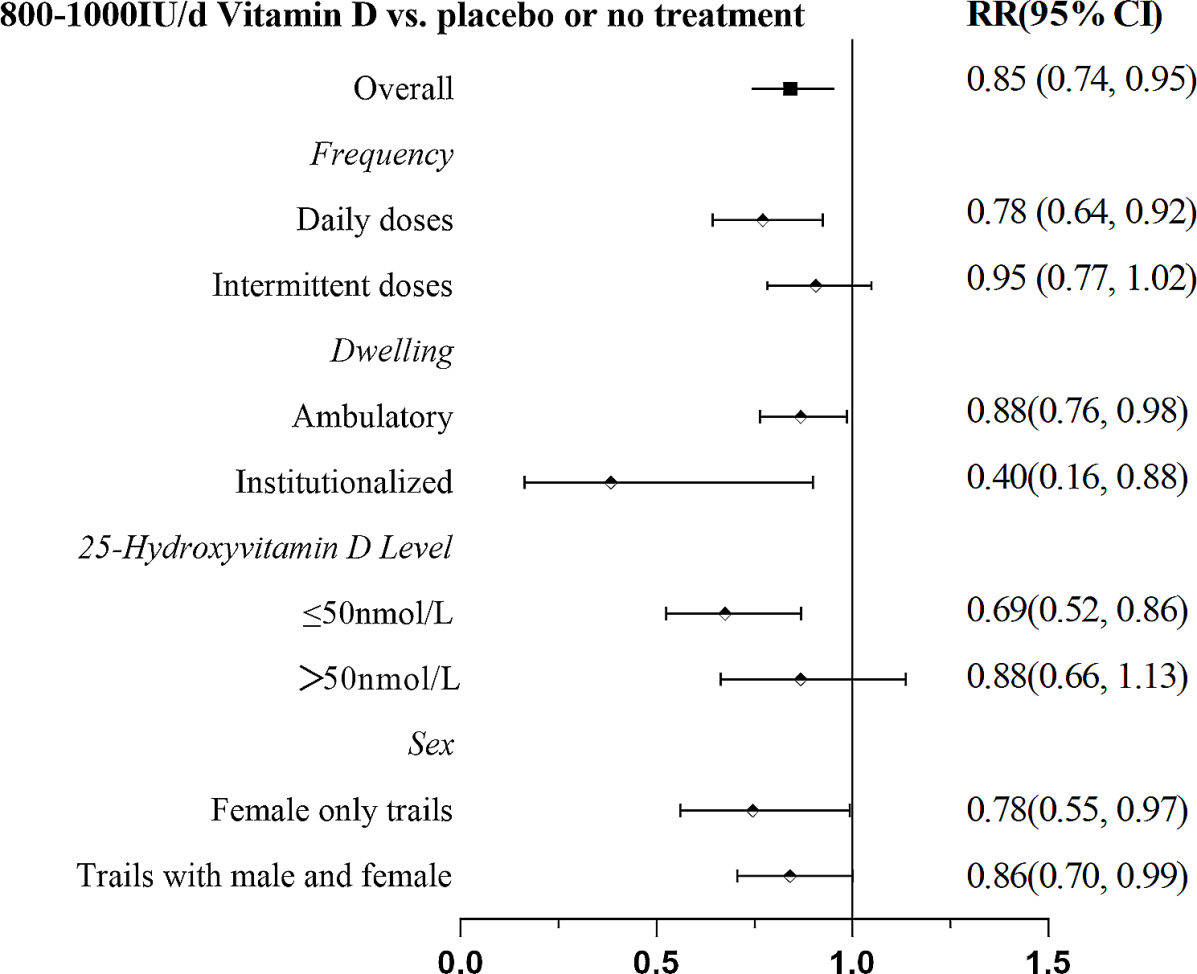
Subgroup analysis of 800–1000 IU/d of vitamin D versus placebo or no treatment

Collectively, our data demonstrated that 800–1000 IU/d of vitamin D was associated with a significantly lower risk of falls in all subgroups except in populations with serum 25(OH)D levels > 50 nmol/L and receiving intermittent doses of vitamin D (Fig. 8).

#### Sensitivity analysis

Fifteen studies with high risk of bias were removed from the sensitivity analysis. NMA was performed on 15 studies since five studies were unable to form a network structure with other studies. The results remained robust in the sensitivity analysis (Table S10 and Fig. S9).

## Discussion

Our NMA of 35 RCTs involving 58,937 elderly individuals demonstrated that vitamin D supplementation at 800–1000 IU/d significantly lowered the incidence of falls by 15%, and the results were robust in the sensitivity analysis. In addition, 800-1000IU/d of vitamin D was associated with a significantly lower risk of falls regardless of gender composition and dwelling. This reduction remained significant in population with vitamin deficiency, while any dose of vitamin D had no effect in older adults with baseline 25(OH)D levels > 50 nmol/L. In terms of dosing frequency, daily administration of 800–1000 IU/d vitamin D reduced the risk of falls by 22%, whereas intermittent administration of vitamin D had no preventive effect on falls (Fig. 8).

Our results are consistent with a previous meta-analysis by Kong et al. [12], in which 800–1000 IU/d of vitamin D was found to be associated with lower risks of falls. Our results are also in line with other meta-analyses indicating that vitamin D can lower the risk of falls despite differences in the recommended doses. The doses that were proposed for fall prevention in these meta-analyses were 700–1000 IU/d of vitamin D by Bischoff-Ferrari et al. [2], 700–2000 IU/d of vitamin D by Wei et al. [14], and 700-1000IU/d vitamin D by Ling et al. [13] According to Thanapluetiwong et al. [11], vitamin D3 decreased the incidence of falls only when supplemented with calcium (RR = 0.881, 95% CI 0.821–0.945), but subgroup analyses were not performed on the different vitamin D doses. Due to differences in the preferred doses of vitamin D in the included studies, we divided vitamin D use into different dose groups and conducted a NMA to test the effectiveness of different dose ranges. We found that only 800–1000 IU/d of vitamin D reduced the risk of falls. Our cumulative probability results based on SUCRA showed that the top three regimens with decreasing effectiveness were 800–1000 IU/d vitamin D alone, 600–700 IU/d vitamin D combined with calcium, and 800–1000 IU/d vitamin D combined with calcium. Our data indicated that 800-1000IU/d of vitamin D was the best dosage for reducing falls. Of note, several meta-analyses indicated that vitamin D decreased the incidence of falls only when supplemented with calcium [10, 11, 13, 14]. In contrast, we found that 800–1000 IU/d vitamin D combined with calcium was only beneficial when compared with calcium alone but not with placebo or no treatment. This discrepancy may be attributed to different control groups. Most of previous meta-analyses used both placebo and calcium alone as the control group, while we assigned placebo and calcium as separate groups. Nevertheless, our results were in agreement with some published recommendations. The BHOF and International Osteoporosis Foundation (IOF) both recommend a daily intake of 800–1000 IU vitamin D for seniors to improve bone health and reduce the risk of falls [6, 60, 61] However, the meta-analysis by Bolland et al. revealed no effect of vitamin D on falls [9], which could be related to the exclusion of studies that compared vitamin D and calcium combined supplementation with placebo. Additionally, the authors did not compare specific dose subgroups with the controls.

High-dose vitamin D application has been shown to be non-beneficial or even harmful. Higher monthly doses of vitamin D (60,000 IU/m) were found to be effective for achieving a serum level of at least 30 ng/mL of 25-hydroxyvitamin D and were associated with an increased risk of falls [52]. Wanigatunga et al. [15] reported that > 1000 IU/d of vitamin D increased the risk of first-time falls with fractures but lowered the risk of outdoor falls in community-dwelling older adults with 25-72.5 nmol/L baseline 25(OH)D level. In our NMA, the incidence of falls did not differ significantly between > 1000 IU/d vitamin D and placebo or no treatment. However, the frequency of falls was significantly higher in the > 1000 IU/d vitamin D groups than in the 800–1000 IU/d group.

Another important finding is that the efficacy of vitamin D in fall prevention depends on the baseline serum level of 25(OH)D in the elderly population and the dosing frequency of vitamin D (daily or intermittent). We found that vitamin D was only beneficial for fall prevention in population with vitamin D deficiency. The US Preventive Services Task Force reported a lack of association between vitamin D supplementation and falls [62] based on only 7 trials, wherein the mean baseline serum 25(OH)D levels ranged from 65.9 to 79.4 nmol/L. Vitamin D supplementation in population without vitamin D deficiency did not have meaningful effects on falls. Moreover, intermittent vitamin D supplementation also showed no protective effect on falls, which was consistent with some previous studies [14, 63]. A meta-analysis published in 2023 showed that intermittent or single high-dose vitamin D3 supplementation increased the risk of falls, and the association was close to statistically significant [63]. Therefore, vitamin D supplementation may be helpful for fall prevention in population with vitamin D deficiency, and is more effective when administered daily rather than intermittently.

Previous studies have suggested that low baseline 25(OH)D may contribute to muscle strength decline in the elderly [64] and is associated with lower 6-minute walking test score and weaker strength [65]. The mechanisms by which vitamin D decreases the occurrence of falls can be partially explained by the findings that vitamin D can regulate calcium homeostasis and improve muscle strength and balance, ultimately leading to a reduced risk of falling [26, 66]. Daily vitamin D supplementation at 800 to 1,000 IU consistently demonstrated beneficial effects on strength and balance [67]. Though, a negative effect of 70 µg (2800 IU)/d of vitamin D on muscle strength and physical performance was reported by Bislev et al. [68]. However, the effect of vitamin D remains controversial. Aschauer et al. [69] revealed that neither muscle strength endurance, nor functional mobility were modulated by vitamin D supplementation (800 IU/d vitamin D3, 50,000 IU/month vitamin D3 or nothing). Therefore, even though our data are consistent with some prior studies, the physiology of vitamin D in falls remains unclear and needs further investigation.

Surprisingly, calcium alone without vitamin D resulted in increased frequency of falls compared with placebo or no treatment, and the association was close to statistically significant (RR = 1.26, 95%CI: 0.997–1.62). Calcium supplementation has been considered to be beneficial for the prevention of osteoporosis and fractures [70, 71]. Though, the usefulness of calcium supplementation in prevention of fractures has been questioned [72]. Warensjö et al. [73] found that dietary calcium intake of > 1,137 mg/d could increase the risk of hip fractures in women. Likewise, a meta-analysis reported that calcium supplementation (480–1000 mg/d elemental calcium) may increase the risk of hip fractures [74]. Reid et al. [75] also found that 5,500 women involved in three trials of calcium monotherapy (480–1000 mg/d elemental calcium) exhibited consistent adverse trends in the number of hip fractures (RR = 1.50, 95%CI: 1.06–2.12). However, there was no direct comparison between calcium alone (without vitamin D) and placebo in our meta-analysis, and indirect comparison was performed through the intermediate node of vitamin D plus calcium. Therefore, further RCTs are warranted to elucidate the impact of calcium supplementation without vitamin D on falls.

There are several limitations in our study. First, the small sample size and use of primary outcomes other than fall in some studies may confound the results. Second, the dosage and frequency of vitamin D administration varied greatly among studies. Although we pooled intermittent doses by calculating the average dose per day, this may lead to bias in the findings considering that various dosing regimens may result in different vitamin D status in the body. Also, we divided vitamin D usage into different dose groups based on previous studies and meta-analyses, which might introduce some bias as a result of the method of grouping. Third, studies performed in different dwellings were included in the NMA and hence our results should be interpreted with caution. Fourth, we excluded non-English articles from our literature search, which may cause selection bias. Indeed, our Egger’s test result indicated the presence of publication bias in our study. Last, there are other confounding factors present in this study, such as potential missing data, meta-biases, and heterogeneity of NMA.

## Conclusion

This is the first systematic review and NMA comparing the efficacy of different concentrations of vitamin D, calcium, and combined supplementation in fall prevention. Based on our NMA, 800–1000 IU/d of vitamin D supplement is associated with a lower risk of falling among older adults. Vitamin D is effective for preventing falls in populations on a daily dosing schedule and deficient in vitamin D, but not in populations receiving intermittent dosing schedule or without vitamin D deficiency. Nevertheless, further well-designed RCTs are warranted to confirm these findings.

### Electronic supplementary material

Below is the link to the electronic supplementary material.

Supplementary Material 1

Supplementary Material 2

## Data Availability

All data generated or analyzed during this study are included in this published article and supplementary materials.

## Abbreviations

BHOF: Bone Health and Osteoporosis Foundation
CI: Confidence interval
DIC: Deviation Information Criterion
ESCEO: European Society for Clinical and Economic Aspects of Osteoporosis, Osteoarthritis and Musculoskeletal Diseases
IOM: Institute of Medicine
IOF: International Osteoporosis Foundation
IU: International Unit
MCMC: Markov Chain Monte Carlo
NMA: Network meta-analysis
RCT: Randomized controlled trial
RR: Relative risk
PRISMA: Preferred Reporting Items for Systemic Reviews and Meta-Analyses
SUCRA: Surface under the cumulative ranking curve
