# Supplementary material for: Effect of vitamin D, calcium, or combined supplementation on fall prevention: a systematic review and updated network meta-analysis
Source: BMC Geriatr. 2024 May 2;24:390. doi: 10.1186/s12877-024-05009-x (PMC11064304; doi:10.1186/s12877-024-05009-x)
Supplement: Supplementary file 2 — Supplementary Material 2 [file 12877_2024_5009_MOESM2_ESM.docx]

**Table S2** Literature search strategy.

1.Pubmed

| Search number | Query |
| --- | --- |
| #1 | ("Ergocalciferols"[Mesh]) OR "Vitamin D"[Mesh] |
| #2 | ((((((((vitamin d[Title/Abstract]) OR (ergocalciferols[Title/Abstract])) OR (ergocalciferol[Title/Abstract])) OR (cholecalciferol[Title/Abstract])) OR (calcifediol[Title/Abstract])) OR (25-hydroxyvitamin D[Title/Abstract])) OR (calcitriol[Title/Abstract])) OR (calcidiol[Title/Abstract])) OR (VD[Title/Abstract]) |
| #3 | (("Ergocalciferols"[Mesh]) OR "Vitamin D"[Mesh]) OR (((((((((vitamin d[Title/Abstract]) OR (ergocalciferols[Title/Abstract])) OR (ergocalciferol[Title/Abstract])) OR (cholecalciferol[Title/Abstract])) OR (calcifediol[Title/Abstract])) OR (25-hydroxyvitamin D[Title/Abstract])) OR (calcitriol[Title/Abstract])) OR (calcidiol[Title/Abstract])) OR (VD[Title/Abstract])) |
| #4 | "Accidental Falls"[Mesh] |
| #5 | (((Accidental Fall[Title/Abstract]) OR (Falling[Title/Abstract])) OR (fall[Title/Abstract])) OR (Falls[Title/Abstract]) |
| #6 | ("Accidental Falls"[Mesh]) OR ((((Accidental Fall[Title/Abstract]) OR (Falling[Title/Abstract])) OR (fall[Title/Abstract])) OR (Falls[Title/Abstract])) |
| #7 | "Randomized Controlled Trial" [Publication Type] |
| #8 | (((randomized controlled trial[Title/Abstract]) OR (randomised controlled study[Title/Abstract])) OR (RCT[Title/Abstract])) OR (random*[Title/Abstract]) |
| #9 | ("Randomized Controlled Trial" [Publication Type]) OR ((((randomized controlled trial[Title/Abstract]) OR (randomised controlled study[Title/Abstract])) OR (RCT[Title/Abstract])) OR (random*[Title/Abstract])) |
| #10 | (((("Ergocalciferols"[Mesh]) OR "Vitamin D"[Mesh]) OR (((((((((vitamin d[Title/Abstract]) OR (ergocalciferols[Title/Abstract])) OR (ergocalciferol[Title/Abstract])) OR (cholecalciferol[Title/Abstract])) OR (calcifediol[Title/Abstract])) OR (25-hydroxyvitamin D[Title/Abstract])) OR (calcitriol[Title/Abstract])) OR (calcidiol[Title/Abstract])) OR (VD[Title/Abstract]))) AND (("Accidental Falls"[Mesh]) OR ((((Accidental Fall[Title/Abstract]) OR (Falling[Title/Abstract])) OR (fall[Title/Abstract])) OR (Falls[Title/Abstract])))) AND (("Randomized Controlled Trial" [Publication Type]) OR ((((randomized controlled trial[Title/Abstract]) OR (randomised controlled study[Title/Abstract])) OR (RCT[Title/Abstract])) OR (random*[Title/Abstract]))) |

2.Cochrane

| Search number | Query |
| --- | --- |
| #1 | MeSH descriptor: [Vitamin D] explode all trees |
| #2 | MeSH descriptor: [Ergocalciferols] explode all trees |
| #3 | (vitamin d):ti,ab,kw OR (ergocalciferols):ti,ab,kw OR (ergocalciferol):ti,ab,kw OR (cholecalciferol):ti,ab,kw OR (calcifediol):ti,ab,kw |
| #4 | ("25-hydroxyvitamin-D"):ti,ab,kw OR (calcitriol):ti,ab,kw OR (calcidiol):ti,ab,kw OR (VD):ti,ab,kw |
| #5 | #1 or #2 or #3 or #4 |
| #6 | MeSH descriptor: [Accidental Falls] explode all trees |
| #7 | (Falling):ti,ab,kw OR (Accidental Fall):ti,ab,kw OR (fall):ti,ab,kw OR (falls):ti,ab,kw |
| #8 | #6 or #7 |
| #9 | MeSH descriptor: [Randomized Controlled Trial] explode all trees |
| #10 | (randomized controlled trial):ti,ab,kw OR (randomised controlled study):ti,ab,kw OR (RCT):ti,ab,kw OR (random*):ti,ab,kw |
| #11 | #9 or #10 |
| #12 | #5 and #8 and #11 |

3.Embase

| Search number | Query |
| --- | --- |
| #1 | \| 'ergocalciferol'/exp \| \| --- \| |
| #2 | 'vitamin d'/exp |
| #3 | 'vitamin d':ab,ti OR ergocalciferols:ab,ti OR ergocalciferol:ab,ti OR cholecalciferol:ab,ti OR calcifediol:ab,ti OR '25-hydroxyvitamin d':ab,ti OR calcitriol:ab,ti OR calcidiol:ab,ti OR vd:ab,ti |
| #4 | #1 OR #2 OR #3 |
| #5 | 'falling'/exp |
| #6 | 'accidental fall':ab,ti OR falling:ab,ti OR fall:ab,ti OR falls:ab,ti |
| #7 | #5 OR #6 |
| #8 | 'randomized controlled trial'/exp |
| #9 | 'randomized controlled trial':ab,ti OR 'randomised controlled study':ab,ti OR random*:ab,ti OR rct:ab,ti |
| #10 | #8 OR #9 |
| #11 | #4 AND #7 AND #10 |

4.Web of science

| Search number | Query |
| --- | --- |
| #1 | vitamin d (Topic) OR ergocalciferols (Topic) OR ergocalciferol (Topic) OR cholecalciferol (Topic) OR calcifediol (Topic) OR 25-hydroxyvitamin D (Topic) OR calcitriol (Topic) OR calcidiol (Topic) OR VD (Topic) |
| #2 | Accidental Fall (Topic) OR Falling (Topic) OR Fall (Topic) OR Falls (Topic) |
| #3 | randomized controlled trial (Topic) OR randomised controlled study (Topic) OR RCT (Topic) OR random* (Topic) |
| #4 | #3 AND #2 AND #1 |

**Table S3** Version 2 of the Cochrane risk-of-bias assessment tool for randomised trials.

| **Bias domain and signalling question*** | **Criteria for judging risk of bias** | **Response options** | | |
| --- | --- | --- | --- | --- |
|  |  | **Lower risk** | **Higher risk** | **Other** |
| **Bias arising from the randomisation process** | | | | |
| 1.1 Was the allocation sequence random? | Answer ‘Yes’ if a random component was used in the sequence generation process(e.g. computer-generated random numbers, shuffling cards or envelopes, throwing dice, or drawing lots). | Y/PY | N/PN | NI |
| 1.2 Was the allocation sequence concealed until participants were enrolled and assigned to interventions? | Answer ‘Yes’ if the allocation sequence was concealed until participants were enrolled and assigned to interventions(e.g. the process of allocation was controlled by an external unit or organization). | Y/PY | N/PN | NI |
| 1.3 Did baseline differences between intervention groups suggest a problem with the randomisation process? | Answer ‘No’ if no imbalances were apparent or if any observed imbalances were compatible with chance. Answer ‘Yes’ if there were imbalances that indicate problems with the randomization process. | N/PN | Y/PY | NI |
| Risk-of-bias judgment (low/high/some concerns) | | | | |
| **Bias due to deviations from intended interventions** | | | | |
| 2.1 Were participants aware of their assigned intervention during the trial? | Answer ‘No’ if participants were blinded to group allocation. | N/PN | Y/PY | NI |
| 2.2 Were carers and people delivering the interventions aware of participants’ assigned intervention during the trial? | Answer ‘No’ if carers and people delivering the interventions were blinded to group allocation. | N/PN | Y/PY | NI |
| 2.3 If Y/PY/NI to 2.1 or 2.2: Were there deviations from the intended intervention that arose because of the trial context? | Answer ‘Yes’ or ‘Probably yes’ only if there was evidence that the trial context led to failure to implement the protocol interventions or to implementation of interventions not allowed by the protocol. Answer ‘No’ or ‘Probably no’ if there were changes from assigned intervention that were inconsistent with the trial protocol, but these were consistent with what could occur outside the trial context. | N/PN | Y/PY | NA/NI |
| 2.4 If Y/PY/NI to 2.3: Were these deviations likely to have affected the outcome? | Answer ‘Yes’ if the deviations were likely to affect the outcome. | N/PN | Y/PY | NA/NI |
| 2.5 If Y/PY to 2.4: Were these deviations from intended intervention balanced between groups? | Answer ‘Yes’ if the deviations from intended intervention were balanced between groups. | Y/PY | N/PN | NA/NI |
| 2.6 Was an appropriate analysis used to estimate the effect of assignment to intervention? | Answer ‘Yes’ if the analysis was appropriate(intention-to-treat analyses and modified intention-to-treat analyses). Answer ‘No’ if the analysis was inappropriate(per-protocol analyses and as treated analyses). | Y/PY | N/PN | NI |
| 2.7 If N/PN/NI to 2.6: Was there potential for a substantial impact (on the result) of the failure to analyse participants in the group to which they were randomised? | Answer ‘Yes’ if more than 10% of participants were analysed in the wrong intervention group or excluded from the analysis and imbalanced between groups. | N/PN | Y/PY | NA/NI |
| Risk-of-bias judgment (low/high/some concerns) | |  | | |
| **Bias due to missing outcome data** | | | | |
| 3.1 Were data for this outcome available for all, or nearly all, participants randomised? | Answer ‘Yes’ if there were no missing outcome data, or less than 10% of outcome data were missing. | Y/PY | N/PN | NI |
| 3.2 If N/PN/NI to 3.1: Is there evidence that the result was not biased by missing outcome data? | Answer ‘Yes’ if there was evidence that the result was not biased by missing outcome data: (1) analysis methods that correct for bias; or (2) sensitivity analyses showing that results were little changed under a range of plausible assumptions about the relationship between missingness in the outcome and its true value. | Y/PY | N/PN | NA |
| 3.3 If N/PN to 3.2: Could missingness in the outcome depend on its true value? | Answer ‘Yes’ if loss to follow up, or withdrawal from the study, could be related to participants’ health status. Answer ‘No’ if all missing outcome data occurred for documented reasons that were unrelated to the outcome(for example, failure of a measuring device or interruptions to routine data collection). | N/PN | Y/PY | NA/NI |
| 3.4 If Y/PY/NI to 3.3: Is it likely that missingness in the outcome depended on its true value? | Answering ‘Yes’ if there were differences between intervention groups in the proportions of missing outcome data, or reported reasons for missing outcome date provide evidence that misssingness depends on its true value. Answer ‘No’ if the analysis accounted for participant characteristics that were likely to explain the relationship between missingness in the outcome and its true value. | N/PN | Y/PY | NA/NI |
| Risk-of-bias judgment (low/high/some concerns) | |  | | |
| **Bias in measurement of the outcome** | | | | |
| 4.1 Was the method of measuring the outcome inappropriate? | Answer ‘No’ if the method of recording fall was appropriate, e.g. fall diary was used, fall was reported or recorded at the time of the event, the interval for asking about falls was at least once a month. | N/PN | Y/PY | NI |
| 4.2 Could measurement or ascertainment of the outcome have differed between intervention groups? | Answer ‘No’ if there was no difference of the measurement of the outcome between groups. | N/PN | Y/PY | NI |
| 4.3 If N/PN/NI to 4.1 and 4.2: Were outcome assessors aware of the intervention received by study participants? | Answer ‘No’ if outcome assessors were blinded to intervention status. | N/PN | Y/PY | NI |
| 4.4 If Y/PY/NI to 4.3: Could assessment of the outcome have been influenced by knowledge of intervention received? | Answer ‘Yes’ if the outcome could be influenced by knowledge of intervention received. Knowledge of the assigned intervention could influence participant-reported outcomes (such as level of pain). They are unlikely to influence observer-reported outcomes that do not involve judgement. | N/PN | Y/PY | NA/NI |
| 4.5 If Y/PY/NI to 4.4: Is it likely that assessment of the outcome was influenced by knowledge of intervention received? | Answer ‘Yes’ if it is likely that the outcome was influenced by knowledge of the intervention received. | N/PN | Y/PY | NA/NI |
| Risk-of-bias judgment (low/high/some concerns) | |  | | |
| **Bias in selection of the reported result** | | | | |
| 5.1 Were the data that produced this result analysed in accordance with a prespecified analysis plan that was finalised before unblinded outcome data were available for analysis? | Answer ‘Yes’ if the data were analysed in accordance with a prespecified analysis plan that was finalised before unblinded outcome data were available for analysis. Answer ‘No’ if the data were not analysed in accordance with a prespecified analysis plan(e.g. post hoc analysis, data-driven analysis). | Y/PY | N/PN | NI |
| Is the numerical result being assessed likely to have been selected, on the basis of the results, from: | | | | |
| 5.2 ... multiple eligible outcome measurements (eg, scales, definitions, time points) within the outcome domain? | Answer ‘Yes’ if there was clear evidence that a domain was measured in multiple eligible ways, but data for only one or a subset of measures was fully reported (without justification), and the fully reported result was likely to have been selected on the basis of the results. | N/PN | Y/PY | NI |
| 5.3 ... multiple eligible analyses of the data? | Answer ‘Yes’ if there was clear evidence that a measurement was analysed in multiple eligible ways, but data for only one or a subset of analyses was fully reported (without justification), and the fully reported result was likely to have been selected on the basis of the results. | N/PN | Y/PY | NI |
| Risk-of-bias judgment (low/high/some concerns) | | | | |
| **Overall bias** | | | | |
| Risk-of-bias judgment (low/high/some concerns) | | | | |
| Y=yes; PY=probably yes; PN=probably no; N=no; NA=not applicable; NI=no information.  *Signalling questions for bias due to deviations from intended interventions relate to the effect of assignment to intervention.  Note: The criteria is developed according to the guidance document for the RoB 2 tool(https://www.riskofbias.info/) | | | | |

**Table S4** League table of treatment comparisons in studies that applied daily dose regimens.

| Intervention | VD800-1000IU/d | VD600-700IU/d+Ca | VD800-1000IU/d+Ca | Placebo or no treatment | VD≤500IU/d | VD600-700IU/d | VD≥2000IU/d | Ca |
| --- | --- | --- | --- | --- | --- | --- | --- | --- |
| VD800-1000IU/d |  | 1.14 (0.81, 1.65) | 1.19 (0.95, 1.51) | **1.28 (1.09, 1.56)** | **1.35 (1.1, 1.71)** | 1.57 (0.95, 2.49) | **1.6 (1.21, 2.1)** | **1.6 (1.2, 2.23)** |
| VD600-700IU/d+Ca | 0.88 (0.61, 1.23) |  | 1.04 (0.73, 1.45) | 1.12 (0.83, 1.52) | 1.19 (0.83, 1.72) | 1.38 (0.77, 2.35) | 1.4 (0.91, 2.06) | 1.4 (0.95, 2.09) |
| VD800-1000IU/d+Ca | 0.84 (0.66, 1.05) | 0.96 (0.69, 1.36) |  | 1.08 (0.94, 1.28) | 1.14 (0.89, 1.49) | 1.32 (0.79, 2.11) | 1.34 (0.97, 1.81) | **1.34 (1.11, 1.68)** |
| Placebo or no treatment | **0.78 (0.64, 0.92)** | 0.89 (0.66, 1.2) | 0.93 (0.78, 1.07) |  | 1.05 (0.86, 1.3) | 1.23 (0.74, 1.89) | 1.25 (0.93, 1.61) | 1.25 (0.97, 1.62) |
| VD≤500IU/d | **0.74 (0.58, 0.91)** | 0.84 (0.58, 1.21) | 0.88 (0.67, 1.12) | 0.95 (0.77, 1.17) |  | 1.16 (0.71, 1.78) | 1.18 (0.89, 1.5) | 1.19 (0.86, 1.64) |
| VD600-700IU/d | 0.64 (0.4, 1.05) | 0.73 (0.43, 1.3) | 0.76 (0.47, 1.27) | 0.82 (0.53, 1.35) | 0.86 (0.56, 1.4) |  | 1.02 (0.61, 1.71) | 1.02 (0.61, 1.78) |
| VD≥2000IU/d | **0.63 (0.48, 0.83)** | 0.71 (0.49, 1.1) | 0.74 (0.55, 1.03) | 0.8 (0.62, 1.08) | 0.84 (0.67, 1.13) | 0.98 (0.59, 1.63) |  | 1 (0.71, 1.49) |
| Ca | **0.63 (0.45, 0.83)** | 0.71 (0.48, 1.05) | **0.74 (0.6, 0.9)** | 0.8 (0.62, 1.03) | 0.84 (0.61, 1.17) | 0.98 (0.56, 1.63) | 1 (0.67, 1.42) |  |

Numbers in each cell represent the RR (95%CI) for falls between the treatment specified in the column versus that specified in the row.

**Table S5** League table of treatment comparisons in studies with ambulatory and community-dwelling elderly.

| Intervention | VD800-1000IU/d | VD600-700IU/d+Ca | VD800-1000IU/d+Ca | VD≤500IU/d | Placebo or no treatment | VD1100-1900IU/d | VD≥2000IU/d | Ca | VD1100-1900IU/d+Ca |
| --- | --- | --- | --- | --- | --- | --- | --- | --- | --- |
| VD800-1000IU/d |  | 1.02 (0.74, 1.45) | 1.03 (0.84, 1.34) | 1.13 (0.93, 1.41) | **1.14 (1.02, 1.32)** | 1.2 (0.98, 1.51) | **1.2 (1.03, 1.44)** | **1.4 (1.04, 2.01)** | 1.52 (0.97, 2.52) |
| VD600-700IU/d+Ca | 0.99 (0.69, 1.35) |  | 1.02 (0.71, 1.47) | 1.12 (0.76, 1.62) | 1.13 (0.83, 1.53) | 1.19 (0.82, 1.69) | 1.18 (0.83, 1.66) | 1.38 (0.91, 2.15) | 1.5 (0.87, 2.64) |
| VD800-1000IU/d+Ca | 0.97 (0.75, 1.2) | 0.98 (0.68, 1.41) |  | 1.09 (0.82, 1.46) | 1.1 (0.91, 1.34) | 1.17 (0.88, 1.52) | 1.16 (0.9, 1.48) | **1.36 (1.09, 1.71)** | 1.47 (0.98, 2.25) |
| VD≤500IU/d | 0.88 (0.71, 1.07) | 0.9 (0.62, 1.31) | 0.91 (0.69, 1.22) |  | 1.01 (0.82, 1.25) | 1.06 (0.82, 1.37) | 1.06 (0.85, 1.33) | 1.24 (0.87, 1.82) | 1.34 (0.82, 2.26) |
| Placebo or no treatment | **0.88 (0.76, 0.98)** | 0.89 (0.65, 1.21) | 0.91 (0.74, 1.1) | 0.99 (0.8, 1.22) |  | 1.06 (0.86, 1.27) | 1.05 (0.89, 1.22) | 1.23 (0.92, 1.68) | 1.33 (0.85, 2.13) |
| VD1100-1900IU/d | 0.83 (0.66, 1.02) | 0.84 (0.59, 1.22) | 0.86 (0.66, 1.14) | 0.94 (0.73, 1.22) | 0.95 (0.79, 1.16) |  | 1 (0.79, 1.27) | 1.16 (0.83, 1.7) | 1.26 (0.78, 2.12) |
| VD≥2000IU/d | **0.83 (0.69, 0.98)** | 0.84 (0.6, 1.2) | 0.86 (0.67, 1.12) | 0.94 (0.75, 1.18) | 0.95 (0.82, 1.12) | 1 (0.79, 1.27) |  | 1.17 (0.85, 1.67) | 1.27 (0.79, 2.09) |
| Ca | **0.71 (0.5, 0.96)** | 0.72 (0.47, 1.1) | **0.74 (0.58, 0.91)** | 0.81 (0.55, 1.15) | 0.81 (0.6, 1.09) | 0.86 (0.59, 1.2) | 0.85 (0.6, 1.18) |  | 1.08 (0.76, 1.53) |
| VD1100-1900IU/d+Ca | 0.66 (0.4, 1.03) | 0.67 (0.38, 1.15) | 0.68 (0.45, 1.02) | 0.75 (0.44, 1.22) | 0.75 (0.47, 1.18) | 0.79 (0.47, 1.28) | 0.79 (0.48, 1.27) | 0.92 (0.65, 1.31) |  |

Numbers in each cell represent the RR (95%CI) for falls between the treatment specified in the column versus that specified in the row.

**Table S6** League table of treatment comparisons in studies with institution-dwelling elderly.

| Intervention | VD800-1000IU/d | VD800-1000IU/d+Ca | VD1100-1900IU/d | VD≥2000IU/d | Placebo or no treatment | VD≤500IU/d | VD1100-1900IU/d+Ca | VD600-700IU/d | Ca |
| --- | --- | --- | --- | --- | --- | --- | --- | --- | --- |
| VD800-1000IU/d |  | 2.2 (0.74, 6.77) | 2.22 (0.54, 7.3) | 2.44 (0.59, 12) | **2.47 (1.14, 6.16)** | **2.71 (1.01, 8.5)** | 2.73 (0.33, 23.15) | 2.91 (0.89, 10.76) | 2.96 (0.54, 17.08) |
| VD800-1000IU/d+Ca | 0.45 (0.15, 1.35) |  | 1.01 (0.23, 3.31) | 1.1 (0.26, 5.45) | 1.1 (0.49, 2.83) | 1.21 (0.39, 4.35) | 1.23 (0.2, 7.39) | 1.3 (0.34, 5.69) | 1.34 (0.36, 5.14) |
| VD1100-1900IU/d | 0.45 (0.14, 1.85) | 0.99 (0.3, 4.34) |  | 1.09 (0.27, 6.76) | 1.08 (0.49, 3.68) | 1.19 (0.4, 5.59) | 1.22 (0.15, 13.38) | 1.29 (0.35, 7.37) | 1.33 (0.23, 10.24) |
| VD≥2000IU/d | 0.41 (0.08, 1.7) | 0.91 (0.18, 3.91) | 0.92 (0.15, 3.69) |  | 1.01 (0.28, 3.51) | 1.1 (0.24, 5.1) | 1.11 (0.1, 11.08) | 1.18 (0.22, 6.48) | 1.21 (0.15, 8.69) |
| Placebo or no treatment | **0.4 (0.16, 0.88)** | 0.91 (0.35, 2.03) | 0.93 (0.27, 2.03) | 0.99 (0.28, 3.52) |  | 1.09 (0.47, 2.6) | 1.11 (0.14, 7.77) | 1.18 (0.38, 3.72) | 1.2 (0.23, 5.66) |
| VD≤500IU/d | **0.37 (0.12, 0.99)** | 0.83 (0.23, 2.56) | 0.84 (0.18, 2.5) | 0.91 (0.2, 4.13) | 0.92 (0.38, 2.13) |  | 1.01 (0.11, 8.2) | 1.08 (0.33, 3.46) | 1.1 (0.17, 6.2) |
| VD1100-1900IU/d+Ca | 0.37 (0.04, 3.02) | 0.81 (0.14, 4.91) | 0.82 (0.07, 6.51) | 0.9 (0.09, 10.11) | 0.9 (0.13, 6.99) | 0.99 (0.12, 9.34) |  | 1.06 (0.12, 11.19) | 1.09 (0.33, 3.69) |
| VD600-700IU/d | 0.34 (0.09, 1.12) | 0.77 (0.18, 2.94) | 0.78 (0.14, 2.87) | 0.85 (0.15, 4.62) | 0.85 (0.27, 2.66) | 0.93 (0.29, 3.04) | 0.94 (0.09, 8.66) |  | 1.02 (0.14, 6.73) |
| Ca | 0.34 (0.06, 1.87) | 0.75 (0.19, 2.8) | 0.75 (0.1, 4.28) | 0.82 (0.12, 6.61) | 0.83 (0.18, 4.29) | 0.91 (0.16, 5.81) | 0.92 (0.27, 3.07) | 0.98 (0.15, 7.21) |  |

Numbers in each cell represent the RR (95%CI) for falls between the treatment specified in the column versus that specified in the row.

**Table S7** League table of treatment comparisons in studies with average baseline 25-hydroxyvitamin D concentration ≤50nmol/L.

| Intervention | VD800-1000IU/d | VD800-1000IU/d+Ca | VD≥2000IU/d | Placebo or no treatment | VD1100-1900IU/d | VD≤500IU/d | VD600-700IU/d | Ca |
| --- | --- | --- | --- | --- | --- | --- | --- | --- |
| VD800-1000IU/d |  | **1.39 (1.02, 1.98)** | **1.44 (1.03, 2.08)** | **1.45 (1.16, 1.94)** | **1.48 (1.09, 2)** | **1.5 (1.13, 2.05)** | **1.75 (1.03, 2.96)** | **1.77 (1.18, 2.94)** |
| VD800-1000IU/d+Ca | **0.72 (0.5, 0.98)** |  | 1.03 (0.67, 1.58) | 1.04 (0.84, 1.36) | 1.07 (0.74, 1.45) | 1.08 (0.75, 1.56) | 1.25 (0.7, 2.19) | 1.27 (0.97, 1.78) |
| VD≥2000IU/d | **0.7 (0.48, 0.97)** | 0.97 (0.63, 1.48) |  | 1.02 (0.71, 1.47) | 1.04 (0.67, 1.53) | 1.04 (0.68, 1.59) | 1.21 (0.66, 2.21) | 1.23 (0.75, 2.14) |
| Placebo or no treatment | **0.69 (0.52, 0.86)** | 0.96 (0.73, 1.19) | 0.98 (0.68, 1.4) |  | 1.03 (0.76, 1.24) | 1.03 (0.76, 1.35) | 1.2 (0.7, 1.95) | 1.22 (0.84, 1.81) |
| VD1100-1900IU/d | **0.67 (0.5, 0.91)** | 0.93 (0.69, 1.35) | 0.96 (0.65, 1.49) | 0.97 (0.81, 1.32) |  | 1.01 (0.75, 1.43) | 1.17 (0.68, 2.06) | 1.18 (0.81, 2.01) |
| VD≤500IU/d | **0.67 (0.49, 0.88)** | 0.93 (0.64, 1.34) | 0.96 (0.63, 1.46) | 0.97 (0.74, 1.31) | 0.99 (0.7, 1.33) |  | 1.16 (0.7, 1.91) | 1.18 (0.75, 1.97) |
| VD600-700IU/d | **0.57 (0.34, 0.98)** | 0.8 (0.46, 1.42) | 0.82 (0.45, 1.52) | 0.83 (0.51, 1.43) | 0.85 (0.49, 1.47) | 0.86 (0.52, 1.44) |  | 1.02 (0.56, 1.98) |
| Ca | **0.57 (0.34, 0.85)** | 0.79 (0.56, 1.03) | 0.81 (0.47, 1.33) | 0.82 (0.55, 1.19) | 0.84 (0.5, 1.24) | 0.85 (0.51, 1.33) | 0.98 (0.51, 1.8) |  |

Numbers in each cell represent the RR (95%CI) for falls between the treatment specified in the column versus that specified in the row.

**Table S8** League table of treatment comparisons in studies that only included female.

Numbers in each cell represent the RR (95%CI) for falls between the treatment specified in the column versus that specified in the row.

| Intervention | VD800-1000IU/d | VD800-1000IU/d+Ca | Placebo or no treatment | VD≥2000IU/d | VD≤500IU/d | VD1100-1900IU/d | Ca | VD1100-1900IU/d+Ca |
| --- | --- | --- | --- | --- | --- | --- | --- | --- |
| VD800-1000IU/d |  | 1.2 (0.89, 1.81) | **1.28 (1.03, 1.81)** | 1.02 (0.57, 1.85) | 1.29 (0.78, 2.25) | 1.38 (0.89, 2.54) | **1.52 (1.02, 2.79)** | 1.65 (0.91, 3.68) |
| VD800-1000IU/d+Ca | 0.84 (0.55, 1.13) |  | 1.07 (0.85, 1.39) | 0.85 (0.44, 1.53) | 1.08 (0.59, 1.86) | 1.16 (0.71, 1.95) | 1.27 (0.94, 1.88) | 1.37 (0.8, 2.62) |
| Placebo or no treatment | **0.78 (0.55, 0.97)** | 0.93 (0.72, 1.18) |  | 0.79 (0.43, 1.36) | 1.01 (0.58, 1.64) | 1.08 (0.69, 1.69) | 1.18 (0.81, 1.87) | 1.28 (0.7, 2.52) |
| VD≥2000IU/d | 0.98 (0.54, 1.76) | 1.18 (0.65, 2.26) | 1.27 (0.73, 2.34) |  | 1.27 (0.59, 2.71) | 1.37 (0.7, 2.91) | 1.5 (0.78, 3.28) | 1.63 (0.74, 4.14) |
| VD≤500IU/d | 0.77 (0.44, 1.28) | 0.93 (0.54, 1.68) | 0.99 (0.61, 1.73) | 0.79 (0.37, 1.68) |  | 1.07 (0.56, 2.21) | 1.18 (0.64, 2.46) | 1.28 (0.59, 3.13) |
| VD1100-1900IU/d | 0.73 (0.39, 1.12) | 0.86 (0.51, 1.41) | 0.92 (0.59, 1.44) | 0.73 (0.34, 1.44) | 0.93 (0.45, 1.77) |  | 1.09 (0.62, 2.09) | 1.18 (0.56, 2.68) |
| Ca | **0.66 (0.36, 0.98)** | 0.79 (0.53, 1.06) | 0.85 (0.53, 1.23) | 0.67 (0.3, 1.28) | 0.85 (0.41, 1.56) | 0.92 (0.48, 1.61) |  | 1.09 (0.66, 1.76) |
| VD1100-1900IU/d+Ca | 0.61 (0.27, 1.1) | 0.73 (0.38, 1.26) | 0.78 (0.4, 1.43) | 0.61 (0.24, 1.36) | 0.78 (0.32, 1.68) | 0.84 (0.37, 1.78) | 0.92 (0.57, 1.51) |  |

**Table S9** League table of treatment comparisons in studies that included both male and female.

| Intervention | VD800-1000IU/d | VD600-700IU/d+Ca | Placebo or no treatment | VD1100-1900IU/d | VD≤500IU/d | VD≥2000IU/d | VD600-700IU/d |
| --- | --- | --- | --- | --- | --- | --- | --- |
| VD800-1000IU/d |  | 1.04 (0.73, 1.57) | **1.16 (1.01, 1.42)** | 1.17 (0.93, 1.53) | 1.2 (0.98, 1.55) | **1.25 (1.05, 1.55)** | 1.46 (0.88, 2.37) |
| VD600-700IU/d+Ca | 0.97 (0.64, 1.38) |  | 1.12 (0.8, 1.59) | 1.13 (0.75, 1.69) | 1.16 (0.78, 1.74) | 1.2 (0.82, 1.75) | 1.4 (0.77, 2.5) |
| Placebo or no treatment | **0.86 (0.7, 0.99)** | 0.89 (0.63, 1.25) |  | 1.01 (0.8, 1.23) | 1.03 (0.84, 1.27) | 1.07 (0.92, 1.25) | 1.25 (0.75, 1.97) |
| VD1100-1900IU/d | 0.85 (0.65, 1.07) | 0.88 (0.59, 1.34) | 0.99 (0.81, 1.24) |  | 1.02 (0.8, 1.35) | 1.06 (0.83, 1.38) | 1.24 (0.73, 2.04) |
| VD≤500IU/d | 0.83 (0.65, 1.02) | 0.86 (0.57, 1.28) | 0.97 (0.79, 1.19) | 0.98 (0.74, 1.26) |  | 1.04 (0.82, 1.29) | 1.21 (0.73, 1.9) |
| VD≥2000IU/d | **0.8 (0.64, 0.95)** | 0.83 (0.57, 1.21) | 0.93 (0.8, 1.09) | 0.94 (0.72, 1.21) | 0.96 (0.77, 1.21) |  | 1.17 (0.7, 1.87) |
| VD600-700IU/d | 0.68 (0.42, 1.13) | 0.71 (0.4, 1.31) | 0.8 (0.51, 1.33) | 0.81 (0.49, 1.37) | 0.82 (0.53, 1.37) | 0.86 (0.53, 1.44) |  |

Numbers in each cell represent the RR (95%CI) for falls between the treatment specified in the column versus that specified in the row.

**Table S10** League table of treatment comparisons in the sensitivity analysis.

| Intervention | VD800-1000IU/d | VD600-700IU/d+Ca | VD≤500IU/d | placebo | VD1100-1900IU/d | VD≥2000IU/d | VD600-700IU/d |
| --- | --- | --- | --- | --- | --- | --- | --- |
| VD800-1000IU/d |  | 1.18 (0.87, 1.72) | **1.31 (1.09, 1.65)** | **1.33 (1.14, 1.63)** | **1.38 (1.14, 1.71)** | **1.42 (1.18, 1.74)** | 1.57 (0.97, 2.46) |
| VD600-700IU/d+Ca | 0.84 (0.58, 1.15) |  | 1.1 (0.78, 1.56) | 1.12 (0.84, 1.5) | 1.17 (0.82, 1.58) | 1.2 (0.83, 1.65) | 1.32 (0.75, 2.2) |
| VD≤500IU/d | **0.76 (0.61, 0.92)** | 0.91 (0.64, 1.28) |  | 1.02 (0.85, 1.23) | 1.05 (0.83, 1.29) | 1.09 (0.86, 1.32) | 1.2 (0.74, 1.82) |
| placebo | **0.75 (0.61, 0.88)** | 0.89 (0.67, 1.19) | 0.98 (0.81, 1.18) |  | 1.04 (0.87, 1.17) | 1.07 (0.88, 1.25) | 1.18 (0.72, 1.79) |
| VD1100-1900IU/d | **0.72 (0.58, 0.88)** | 0.86 (0.63, 1.22) | 0.95 (0.78, 1.2) | 0.96 (0.85, 1.15) |  | 1.03 (0.83, 1.27) | 1.14 (0.69, 1.78) |
| VD≥2000IU/d | **0.7 (0.58, 0.85)** | 0.83 (0.61, 1.2) | 0.92 (0.76, 1.16) | 0.94 (0.8, 1.14) | 0.97 (0.79, 1.21) |  | 1.1 (0.67, 1.74) |
| VD600-700IU/d | 0.64 (0.41, 1.03) | 0.76 (0.45, 1.33) | 0.83 (0.55, 1.35) | 0.85 (0.56, 1.38) | 0.88 (0.56, 1.44) | 0.91 (0.57, 1.49) |  |

| Intervention | VD800-1000IU/d | VD600-700IU/d+Ca | VD≤500IU/d | placebo | VD1100-1900IU/d | VD≥2000IU/d | VD600-700IU/d |
| --- | --- | --- | --- | --- | --- | --- | --- |
| VD800-1000IU/d |  | 1.18 (0.87, 1.72) | **1.31 (1.09, 1.65)** | **1.33 (1.14, 1.63)** | **1.38 (1.14, 1.71)** | **1.42 (1.18, 1.74)** | 1.57 (0.97, 2.46) |
| VD600-700IU/d+Ca | 0.84 (0.58, 1.15) |  | 1.1 (0.78, 1.56) | 1.12 (0.84, 1.5) | 1.17 (0.82, 1.58) | 1.2 (0.83, 1.65) | 1.32 (0.75, 2.2) |
| VD≤500IU/d | **0.76 (0.61, 0.92)** | 0.91 (0.64, 1.28) |  | 1.02 (0.85, 1.23) | 1.05 (0.83, 1.29) | 1.09 (0.86, 1.32) | 1.2 (0.74, 1.82) |
| placebo | **0.75 (0.61, 0.88)** | 0.89 (0.67, 1.19) | 0.98 (0.81, 1.18) |  | 1.04 (0.87, 1.17) | 1.07 (0.88, 1.25) | 1.18 (0.72, 1.79) |
| VD1100-1900IU/d | **0.72 (0.58, 0.88)** | 0.86 (0.63, 1.22) | 0.95 (0.78, 1.2) | 0.96 (0.85, 1.15) |  | 1.03 (0.83, 1.27) | 1.14 (0.69, 1.78) |
| VD≥2000IU/d | **0.7 (0.58, 0.85)** | 0.83 (0.61, 1.2) | 0.92 (0.76, 1.16) | 0.94 (0.8, 1.14) | 0.97 (0.79, 1.21) |  | 1.1 (0.67, 1.74) |
| VD600-700IU/d | 0.64 (0.41, 1.03) | 0.76 (0.45, 1.33) | 0.83 (0.55, 1.35) | 0.85 (0.56, 1.38) | 0.88 (0.56, 1.44) | 0.91 (0.57, 1.49) |  |

Numbers in each cell represent the RR (95%CI) for falls between the treatment specified in the column versus that specified in the row.

| Intervention | VD800-1000IU/d | VD600-700IU/d+Ca | VD≤500IU/d | placebo | VD1100-1900IU/d | VD≥2000IU/d | VD600-700IU/d |
| --- | --- | --- | --- | --- | --- | --- | --- |
| VD800-1000IU/d |  | 1.18 (0.87, 1.72) | **1.31 (1.09, 1.65)** | **1.33 (1.14, 1.63)** | **1.38 (1.14, 1.71)** | **1.42 (1.18, 1.74)** | 1.57 (0.97, 2.46) |
| VD600-700IU/d+Ca | 0.84 (0.58, 1.15) |  | 1.1 (0.78, 1.56) | 1.12 (0.84, 1.5) | 1.17 (0.82, 1.58) | 1.2 (0.83, 1.65) | 1.32 (0.75, 2.2) |
| VD≤500IU/d | **0.76 (0.61, 0.92)** | 0.91 (0.64, 1.28) |  | 1.02 (0.85, 1.23) | 1.05 (0.83, 1.29) | 1.09 (0.86, 1.32) | 1.2 (0.74, 1.82) |
| placebo | **0.75 (0.61, 0.88)** | 0.89 (0.67, 1.19) | 0.98 (0.81, 1.18) |  | 1.04 (0.87, 1.17) | 1.07 (0.88, 1.25) | 1.18 (0.72, 1.79) |
| VD1100-1900IU/d | **0.72 (0.58, 0.88)** | 0.86 (0.63, 1.22) | 0.95 (0.78, 1.2) | 0.96 (0.85, 1.15) |  | 1.03 (0.83, 1.27) | 1.14 (0.69, 1.78) |
| VD≥2000IU/d | **0.7 (0.58, 0.85)** | 0.83 (0.61, 1.2) | 0.92 (0.76, 1.16) | 0.94 (0.8, 1.14) | 0.97 (0.79, 1.21) |  | 1.1 (0.67, 1.74) |
| VD600-700IU/d | 0.64 (0.41, 1.03) | 0.76 (0.45, 1.33) | 0.83 (0.55, 1.35) | 0.85 (0.56, 1.38) | 0.88 (0.56, 1.44) | 0.91 (0.57, 1.49) |  |


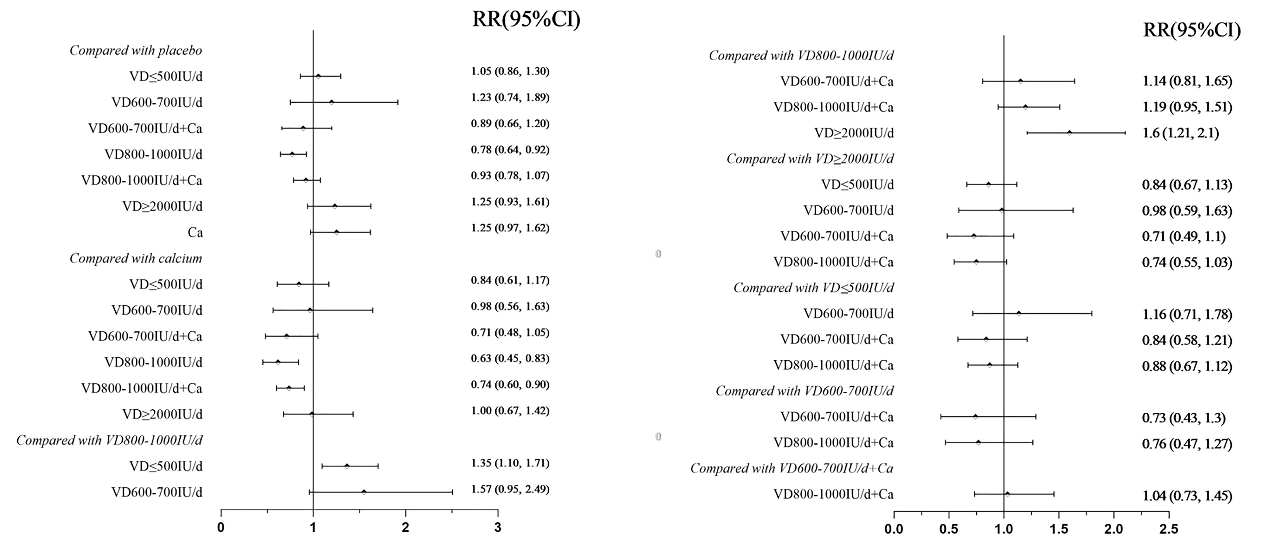
**Fig.S1** The forest plot for the risk of falls in studies that applied daily dose regimens.


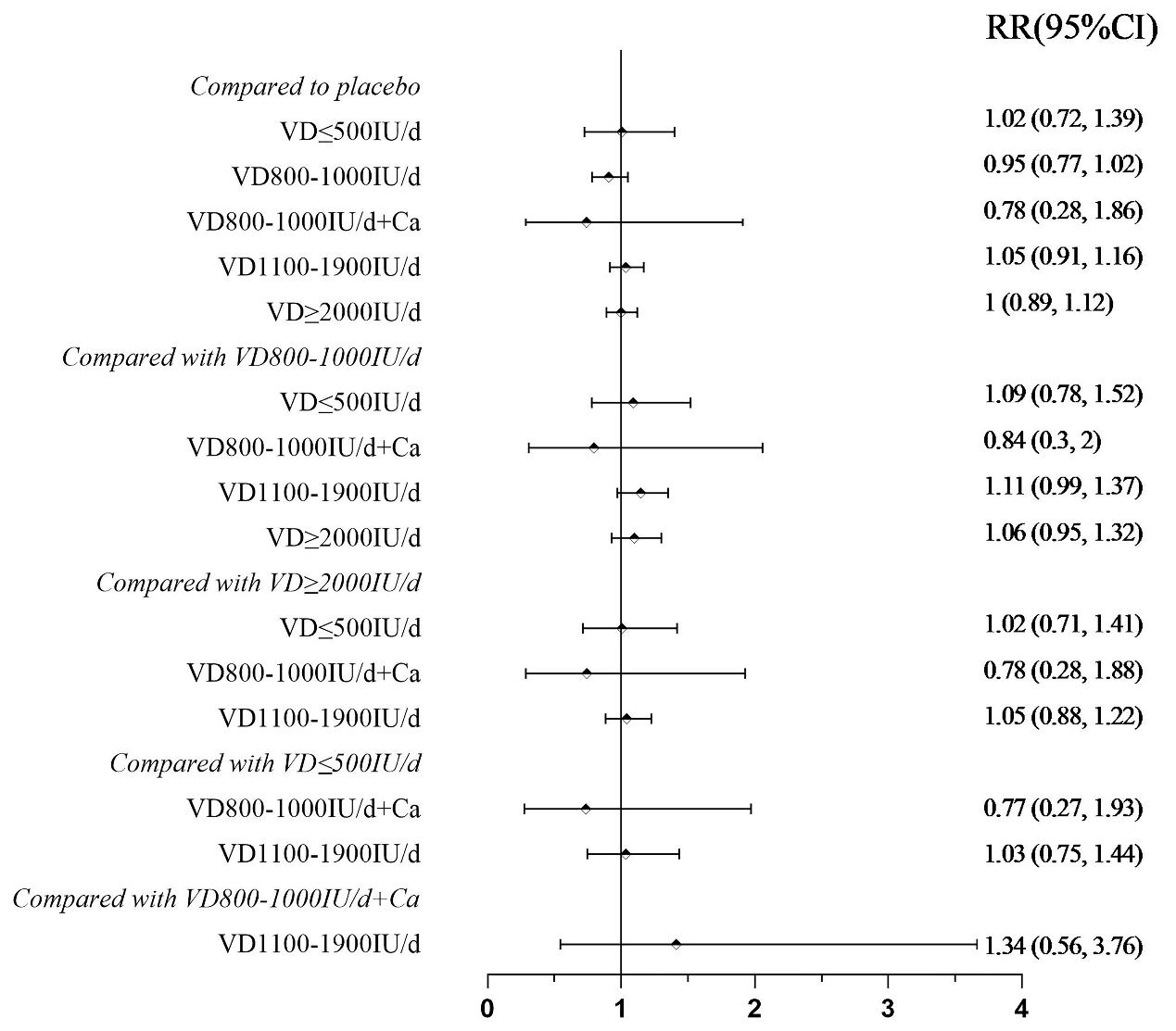


**Fig.S2** The forest plot for the risk of falls in studies that applied intermittent dose regimens.


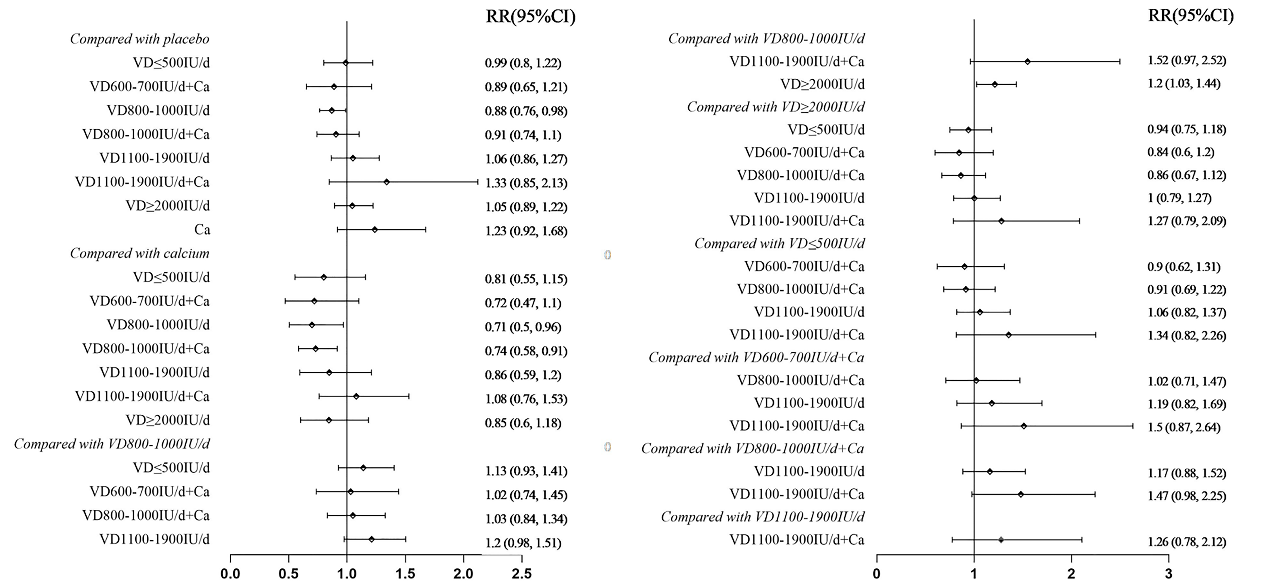


**Fig.S3** The forest plot for the risk of falls in studies with ambulatory and community-dwelling elderly.


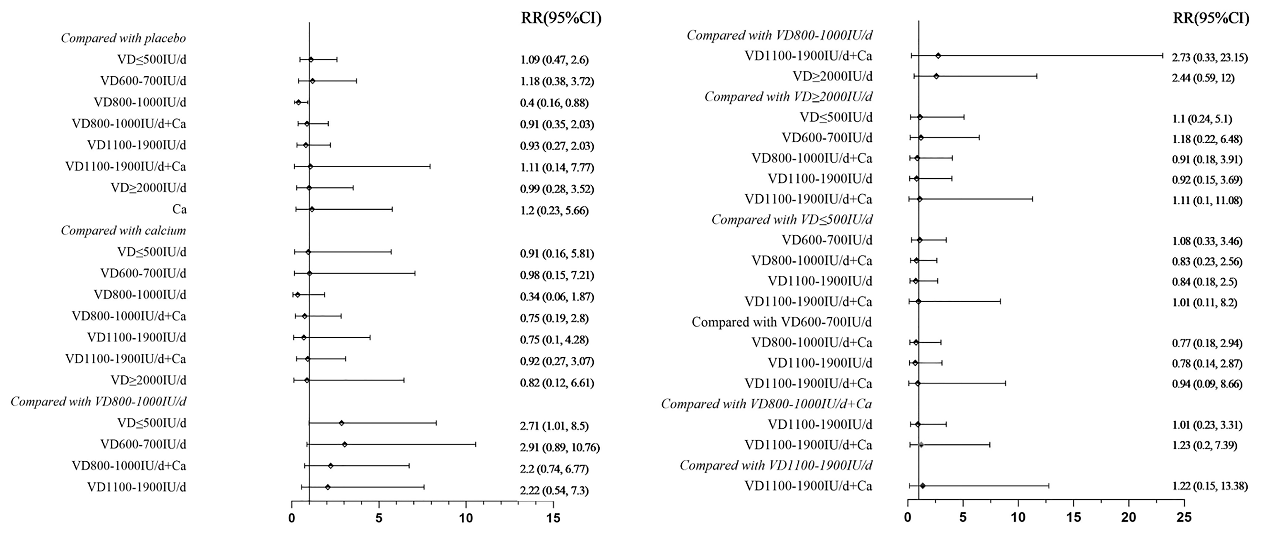


**Fig.S4** The forest plot for the risk of falls in studies with institution-dwelling elderly.


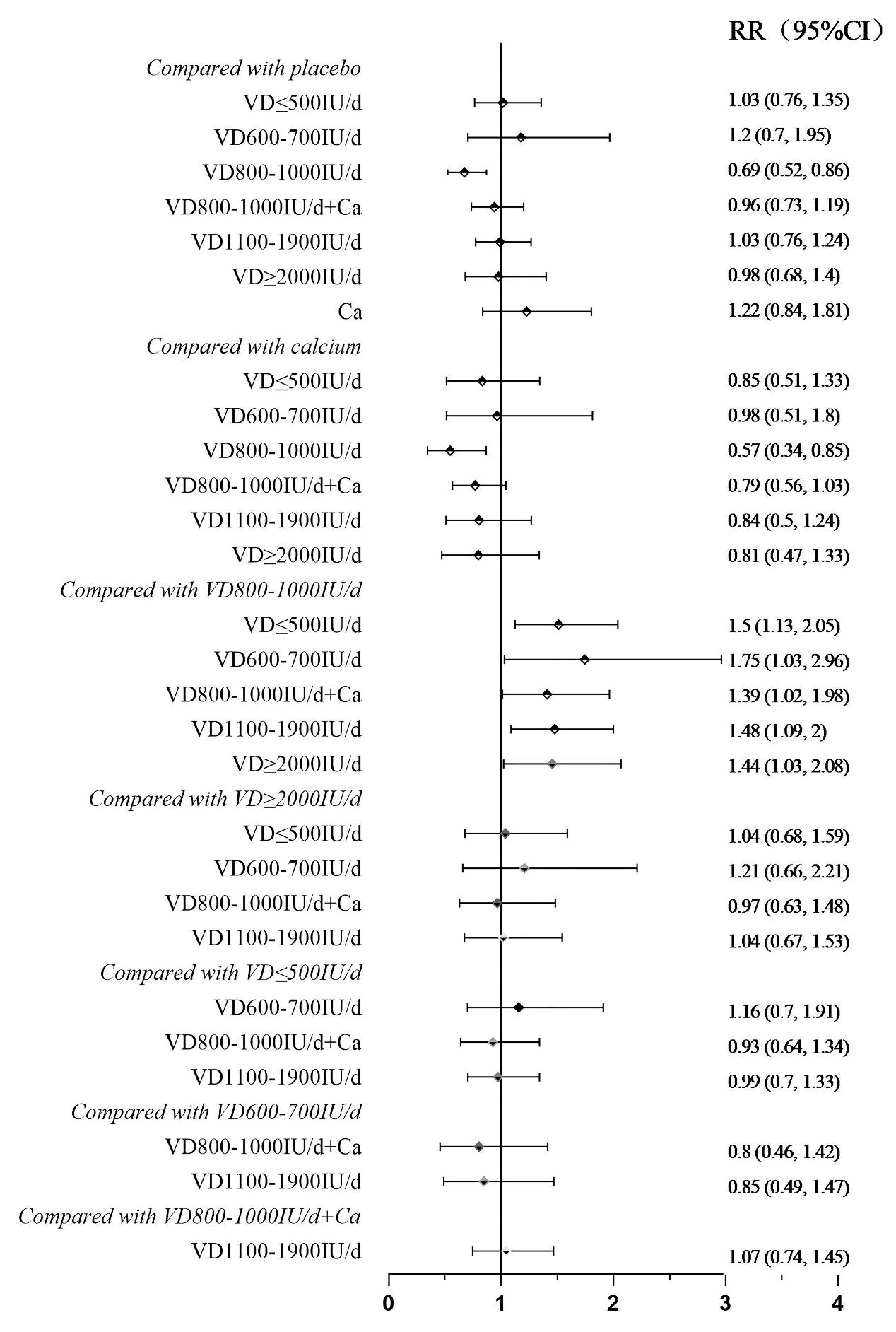
**Fig.S5** The forest plot for the risk of falls in studies with average baseline 25(OH)D concentration ≤50nmol/L.


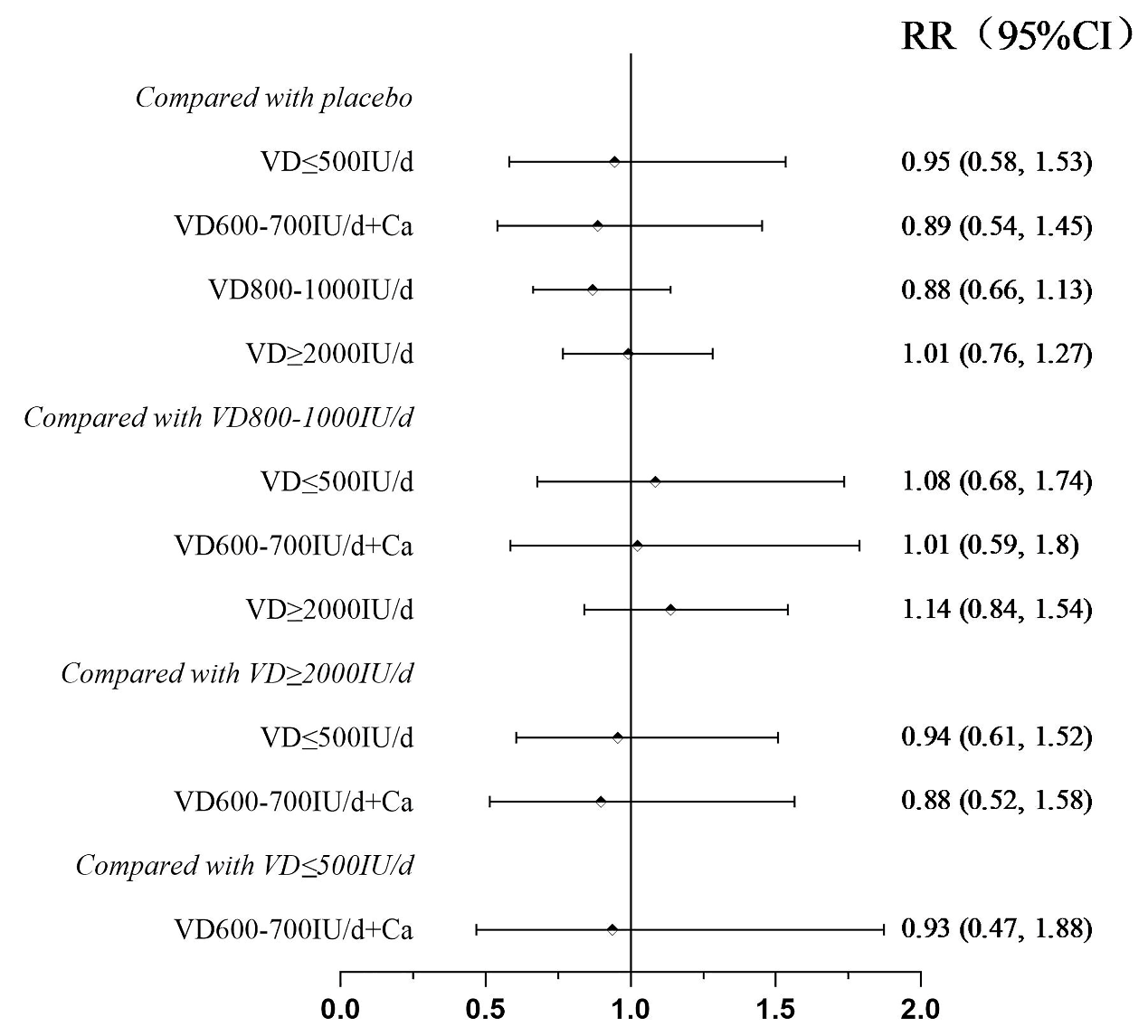


**Fig.S6** The forest plot for the risk of falls in studies with average baseline 25(OH)D concentration＞50nmol/L.


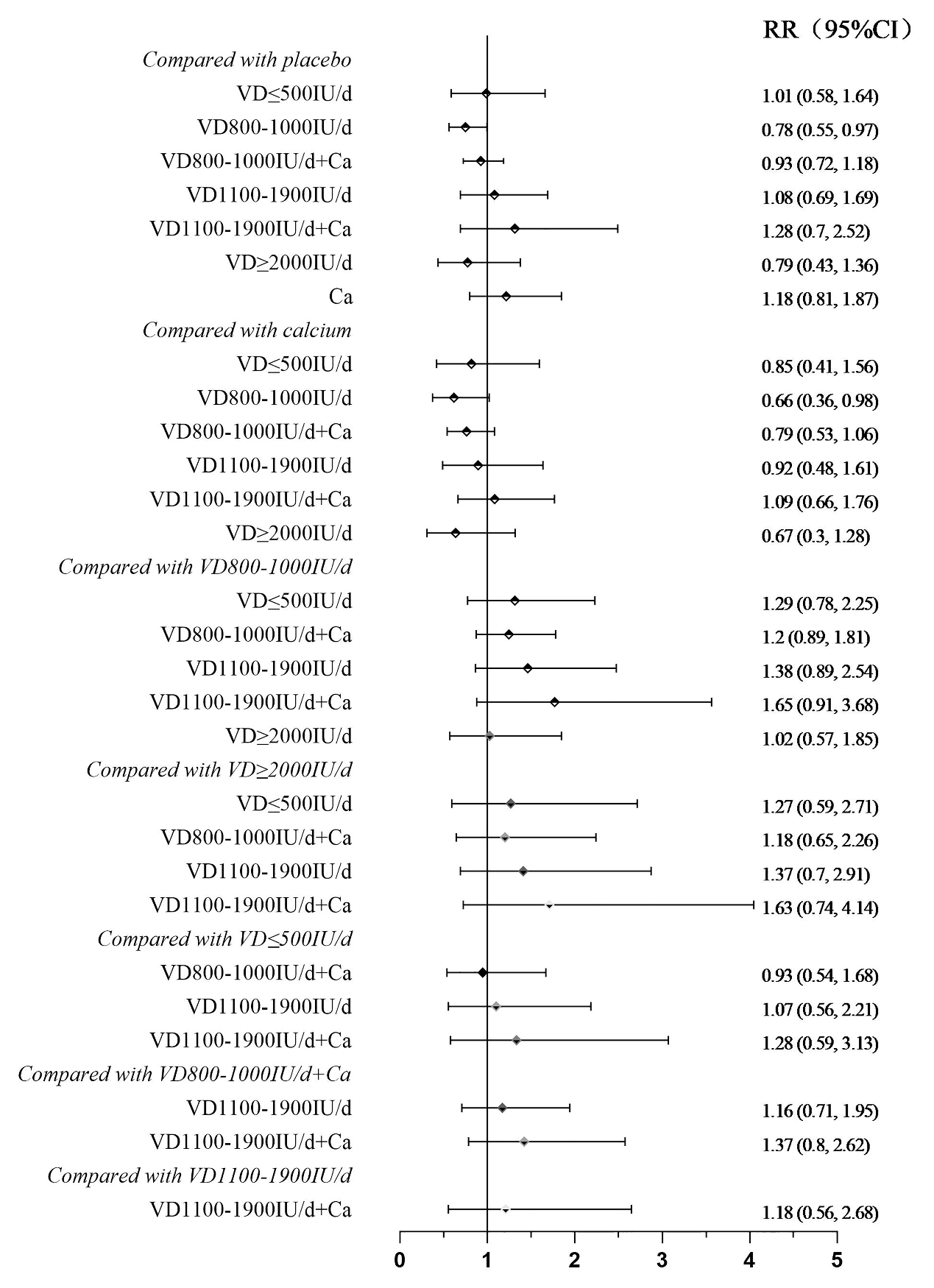
**Fig.S7** The forest plot for the risk of falls in studies only included female.


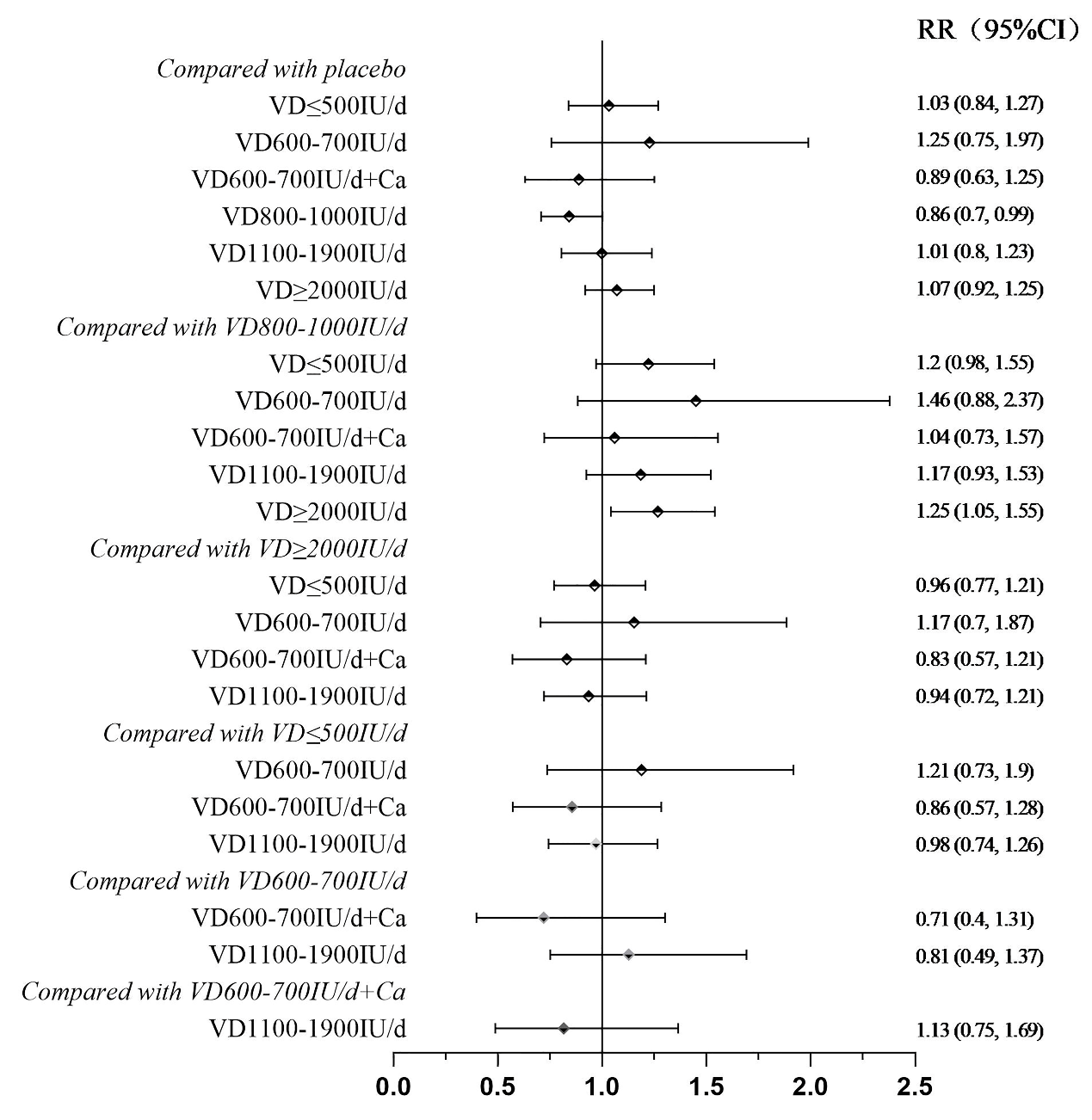


**Fig.S8** The forest plot for the risk of falls in studies included both male and female.


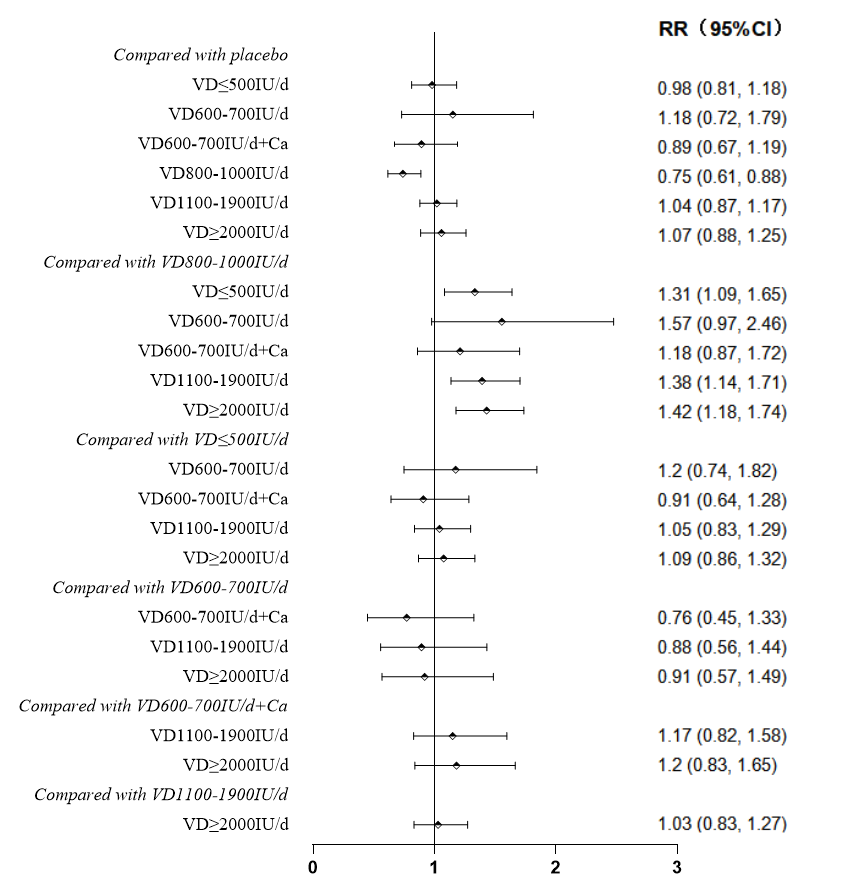


**Fig.S9** The forest plot for the risk of falls in the sensitivity analysis.
